# Simultaneous Quantification of Nine Target Compounds in Traditional Korean Medicine, Bopyeo-Tang, Using High-Performance Liquid Chromatography–Photodiode Array Detector and Ultra-Performance Liquid Chromatography–Tandem Mass Spectrometry

**DOI:** 10.3390/molecules29051171

**Published:** 2024-03-06

**Authors:** Chang-Seob Seo

**Affiliations:** KM Science Research Division, Korea Institute of Oriental Medicine, Daejeon 34054, Republic of Korea; csseo0914@kiom.re.kr; Tel.: +82-42-868-9361

**Keywords:** simultaneous quantification, traditional Korean medicine, Bopyeo-tang, HPLC–PDA, UPLC–MS/MS

## Abstract

Bopyeo-tang (BPT) is composed of six medicinal herbs (*Morus alba* L., *Rehmannia glutinosa* (Gaertn.) DC., *Panax ginseng* C.A.Mey., *Aster tataricus* L.f., *Astragalus propinquus* Schischkin, and *Schisandra chinensis* (Turcz.) Baill.) and has been used for the treatment of lung diseases. This study focused on establishing an analytical method that can simultaneously quantify nine target compounds (i.e., hydroxymethylfurfural, mulberroside A, chlorogenic acid, calycosin-7-*O*-glucoside, 3,5-dicaffeoylquinic acid, quercetin, kaempferol, schizandrin, and gomisin A) from a BPT sample using high-performance liquid chromatography with a photodiode array detector (HPLC–PDA) and ultra-performance liquid chromatography with tandem mass spectrometry (UPLC–MS/MS). The separation of compounds in both analyses was performed on a C_18_ reversed-phase column using the gradient elution of water–acetonitrile as the mobile phase. In particular, the multiple reaction monitoring mode was applied for quick and accurate detection in UPLC–MS/MS analysis. As a result of analyzing the two methods, HPLC–PDA and UPLC–MS/MS, the coefficient of determination of the regression equation for each compound was ≥0.9952, and recovery was 85.99−106.40% (relative standard deviation (RSD) < 9.58%). Precision testing of the nine compounds was verified (RSD < 10.0%). The application of these analytical assays under optimized conditions for quantitative analysis of the BPT sample gave 0.01–4.70 mg/g. Therefore, these two assays could be used successfully to gather basic data for clinical research and the quality control of BPT.

## 1. Introduction

Traditional herbal medicine prescriptions using various combinations of at least two or more herbal medicines have long been used for the treatment or prevention of diseases, due to their multicomponent and multitarget characteristics [1,2,3]. Bopyeo-tang (BPT, Bufei-tang in Chinese) is a traditional herbal medicine prescription consisting of six medicinal herbs (*Morus alba* L., *Rehmannia glutinosa* (Gaertn.) DC., *Panax ginseng* C.A.Mey., *Aster tataricus* L.f., *Astragalus propinquus* Schischkin, and *Schisandra chinensis* (Turcz.) Baill.) in a ratio of 3:3:1:1:1:1 [4]. BPT has been widely used to treat respiratory diseases, such as lung qi deficiency, particularly in elderly men [5,6]. Among BPT’s herbal ingredients, *M. alba* (moracins, kuwanone E, and kuwanone G), *R. glutinosa* (acteoside), *P. ginseng* (ginsenosides), and *S. chinensis* (schisantherin B) have traditionally been used for diseases related to respiratory inflammation [7]. Aster saponins (especially aster saponin B) and 4-hydroxyphenylacetic acid isolated from *A. tataricus* have shown clinical potential for the treatment of acute lung injury [8,9]. In addition, Yu et al. [10] showed that the therapeutic effect of astragalosides, such as astragaloside I, astragaloside II, and astragaloside IV, isolated from *A. propinquus,* on pulmonary fibrosis is mediated by the Ras–Raf–MEK–ERK signaling pathway. Therefore, BPT has been reported to have therapeutic effects on lung-related diseases, such as pulmonary fibrosis, lung cancer, and chronic obstructive pulmonary disease [5,6,11,12,13].

A standardization study of BPT showing efficacy In lung-related diseases with a complex mechanism was reported in a study conducted using high-performance liquid chromatography–diode array detection–electrospray ionization–hybrid ion trap–time-of-flight mass spectrometry (HPLC–DAD–ESI–IT–TOF–MS) by He et al. [13]. They were the first to perform a chemical profiling analysis of the main components of BPT using the HPLC–DAD–ESI–IT–TOF–MS analytical technique, but no quantitative analysis was reported. However, a number of standardization studies have reported the quality control of each raw herbal medicine constituting BPT using various analytical techniques, such as HPLC and liquid chromatography with tandem mass spectrometry (LC–MS/MS) [14,15,16,17,18,19].

Therefore, in this study, a simultaneous quantification of the nine target compounds (i.e., hydroxymethylfurfural, mulberroside A, chlorogenic acid, calycosin-7-*O*-glucoside, 3,5-dicaffeoylquinic acid, quercetin, kaempferol, schizandrin, and gomisin A) in BPT was performed using HPLC with photodiode array detection (HPLC–PDA) and ultra-performance liquid chromatography with tandem mass spectrometry (UPLC–MS/MS). Common analytical instruments were used.

## 2. Results and Discussion

### 2.1. HPLC–PDA Analysis

#### 2.1.1. Selection of Target Compounds in BPT for Simultaneous Quantification by HPLC–PDA

For the selection of target compounds for the quality assessment of BPT, 17 candidate components were compared with the samples (BPT, *M. alba*, *R. glutinosa*, *P. ginseng*, *A. tataricus*, *A. propinquus*, and *S. chinensis* samples). Specifically, the 17 components to be compared were the following: mulberroside A, rutin, isoquercetin, and resveratrol of *M. alba*; hydroxymethylfurfural of *R. glutinosa*; ginsenoside Rb_1_ and ginsenoside Rg_1_ of *P. ginseng*; chlorogenic acid, 3,4-dicaffeoylquinic acid, 3,5-dicaffeoylquinic acid, quercetin, and kaempferol of *A. tataricus*; astragaloside IV and calycosin-7-*O*-glucoside of *A. propinquus*; and schizandrin, gomisin A, and gomisin N of *S. chinensis*. Comparison HPLC chromatograms for each sample and the candidate components are shown in Figure S1. Following a comparison of the results, among the 17 candidate components, nine compounds were finally detected in the BPT sample, which were then selected as target compounds in BPT for simultaneous quantification by HPLC–PDA.

#### 2.1.2. HPLC Operating Conditions for Simultaneous Quantification of BPT

Various parameters, such as the type of column and the temperature of the column oven, and the acid added to the mobile phase were compared to determine the optimal HPLC analytical conditions for the simultaneous quantification of the nine targets selected (Figure S2) from a BPT sample. As a first step, reverse-phase C_18_ columns from different manufacturers were compared to select an appropriate column for the separation of the target compounds. Columns included the following: SunFire^TM^ (Waters), Capcell Pak UG80 (Shiseido, Tokyo, Japan), and Gemini (Phenomenex, Torrance, CA, USA). The columns were identical in length (250 mm), inner diameter (4.6 mm), and particle size (5 μm). As shown in Figure S3, seven and eight components were detected on the Gemini column (Figure S3B) and the Capcell Pak UG80 column (Figure S3D), respectively, while nine components were detected on the Waters SunFire^TM^ column (Figure S3F). The latter column was therefore considered the most preferable for further work.

As a second step, the effects of the acid(s) (i.e., formic acid, phosphoric acid, trifluoroacetic acid, and acetic acid) added to the mobile phase on the separation of the nine target compounds in the first determined column were compared. As a result, as shown in Figure S4, when trifluoroacetic acid and phosphoric acid were added, the 3,5-dicaffeoylquinic acid of both acids overlapped with the peak of an unknown peak, and it was detected (Figure S4B,D). Also, when acetic acid was added, the calycosin-7-*O*-glucoside overlapped with the unknown peak, and it was detected (Figure S4F). However, in the case of formic acid, the nine target compounds were well separated without interference from neighboring components (Figure S4H). Therefore, formic acid was selected as the acid of choice to be added to the mobile phase. Different column temperatures (30, 35, and 40 °C) were considered, and 30 °C was determined to be the most suitable (Figure S5).

**Figure 1:**
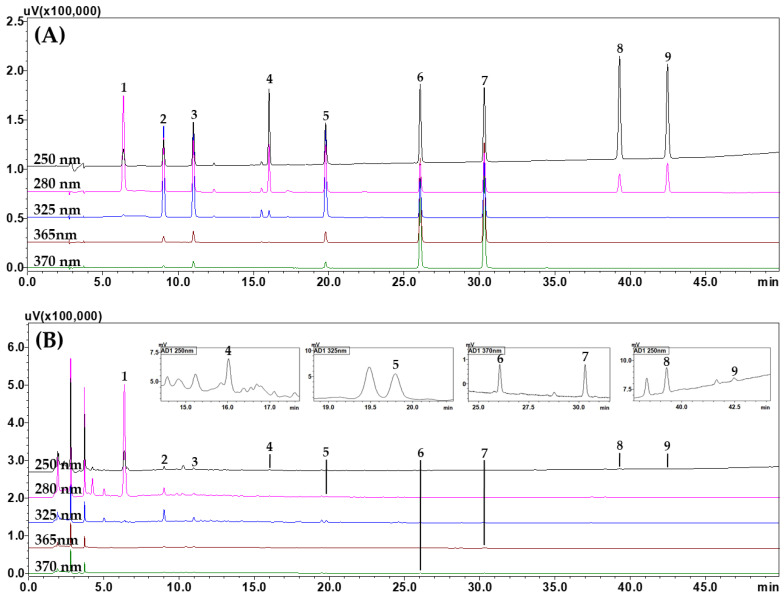
Representative HPLC chromatograms of the mixed standard solution (**A**) and BPT sample (**B**). Hydroxymethylfurfural (1), mulberroside A (2), chlorogenic acid (3), calycosin-7-*O*-glucoside (4), 3,5-dicaffeoylquinic acid (5), quercetin (6), kaempferol (7), schizandrin (8), and gomisin A (9). The concentrations of each compound in the mixed standard solution were as follows: 10.00 μg/mL (hydroxymethylfurfural and calycosin-7-*O*-glucoside), 20.00 μg/mL (chlorogenic acid, 3,5-dicaffeoylquinic acid, quercetin, and kaempferol), 40.00 μg/mL (mulberroside A), and 50.00 μg/mL (schizandrin and gomisin A).

The following were then established as the optimal conditions for the simultaneous analysis of the nine target compounds from a BPT sample: Waters SunFire^TM^ column, distilled water–acetonitrile mobile phase (both containing 0.1% (*v/v*) formic acid), and 30 °C column temperature. Table S1 summarizes the optimized analytical conditions and gradient elution conditions of the mobile phase in more detail. Under the established optimal analysis conditions, all target compounds were completely eluted within 45 min with a resolution of ≥10.70. Representative HPLC chromatograms are shown in Figure 1.

#### 2.1.3. Validation of the Established HPLC–PDA Analytical Method

In the HPLC–PDA method-established simultaneous analysis, the system suitability was confirmed by various parameters, such as the retention factor (1.24–13.98), separation factor (1.09–1.76), theoretical plate number (36,031.97–1201,612.01), resolution (10.70–20.79), and symmetry factor (1.05–1.20) (Table S2). The *r*^2^ values in the calibration curves of each target prepared at different concentrations were ≥0.9999, exhibiting excellent linearity (Table 1). Sensitivities, such as limit of detection (LOD) and limit of quantitation (LOQ), were 0.01–0.08 μg/mL and 0.04–0.26 μg/mL, respectively (Table 1). The recovery test results exhibited accuracy (Table 2). Values for recovery tested using the standard addition method were 95.93%–106.40% (relative standard deviation (RSD, %) ≤ 1.93%). Accuracy evaluation was considered appropriate within the tolerance range of ±20%. Finally, in terms of precision (intra- and inter-day precision and repeatability) evaluated by RSD values, all target compounds had RSD values ≤ 20%, considered an acceptable limit (Table 3 and Table S3).

Suitable results were found for all the verification parameters, which confirmed that the established analytical method was suitable for the simultaneous quantification of the nine target compounds selected from BPT.

#### 2.1.4. Simultaneous Quantification of Nine Target Compounds in a BPT Sample by the HPLC–PDA Analytical Method

The nine selected targets (i.e., hydroxymethylfurfural, mulberroside A, chlorogenic acid, calycosin-7-*O*-glucoside, 3,5-dicaffeoylquinic acid, quercetin, kaempferol, schizandrin, and gomisin A) were simultaneously quantified in BPT using an established HPLC analytical method. Quantification of each target was performed based on the maximum ultraviolet absorption wavelength using a PDA detector, as shown in Table 1. Table 4 shows the quantitative analysis results obtained by applying the optimized HPLC analytical method to the BPT sample. Nine target compounds were detected in 0.01–3.02 mg/freeze-dried g in BPT. Among them, hydroxymethylfurfural and mulberroside A, the main components of *R. glutinosa* and *M. alba*, were found to be abundant (i.e., 3.02 mg/g and 1.81 mg/g, respectively).

### 2.2. UPLC–MS/MS Simultaneous Analysis

#### 2.2.1. UPLC–MS/MS Multiple Reaction Monitoring (MRM) Method for Simultaneous Analysis

**Figure 2:**
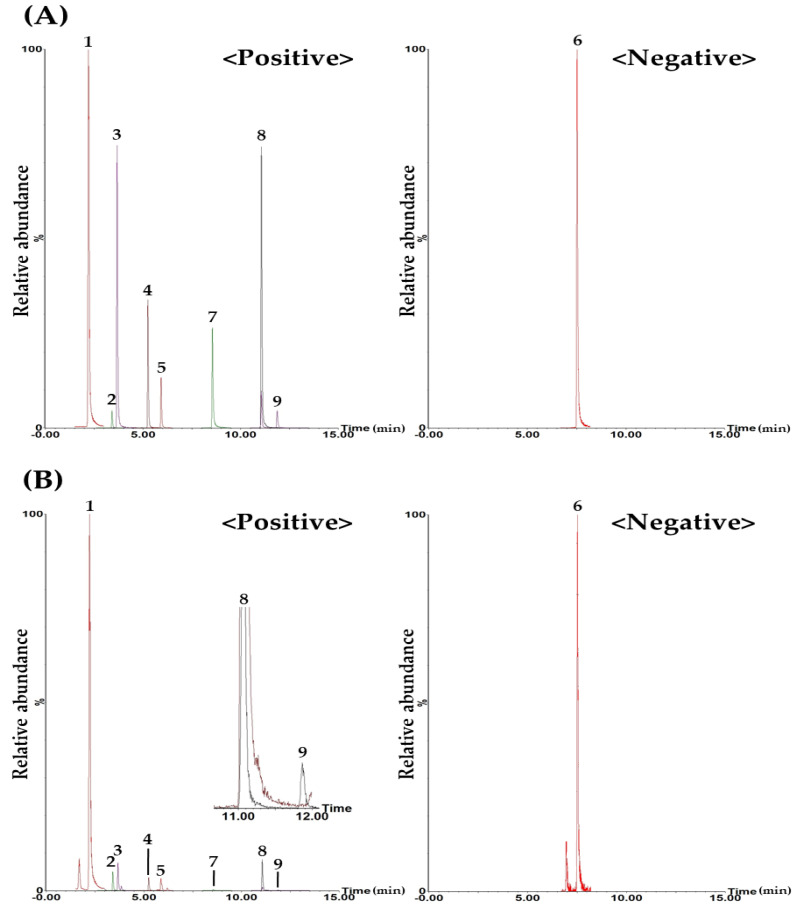
Representative total ion chromatograms of the standard solution (**A**) and the BPT sample (**B**) using the UPLC−MS/MS MRM method. Hydroxymethylfurfural (1), mulberroside A (2), chlorogenic acid (3), calycosin-7-*O*-glucoside (4), 3,5-dicaffeoylquinic acid (5), quercetin (6), kaempferol (7), schizandrin (8), and gomisin A (9). The concentrations of each compound in the mixed standard solution were as follows: 1250.00 μg/L (hydroxymethylfurfural), 250.00 μg/L (mulberroside A), 1000.00 μg/L (chlorogenic acid), 175.00 μg/L (calycosin-7-*O*-glucoside and 3,5-dicaffeoylquinic acid), 750.00 μg/L (quercetin), 1000.00 μg/L (kaempferol), and 375.00 μg/L (schizandrin and gomisin A).

Simultaneous determination of target components in BPT by UPLC–MS/MS was conducted on the nine compounds selected in the HPLC–PDA analysis assay. As a result of detecting these components, using the ESI mode, two components (i.e., chlorogenic acid and quercetin) were detected in negative ion mode, and the other seven components (i.e., hydroxymethylfurfural, mulberroside A, calycosin-7-*O*-glucoside, 3,5-dicaffeoylquinic acid, kaempferol, schizandrin, and gomisin A) were detected in positive ion mode (Figure 2 and Figure S6).

The MRM transitions (precursor ion (Q1) and product ion (Q3)) of each compound are shown in Table 5 and Figure S7. Briefly, hydroxymethylfurfural and schizandrin were set to *m/z* 109.0 and 415.0, which are the ions generated by the removal of a water molecule from Q1, respectively [20,21]. The Q3 peak of mulberroside A was set at *m/z* 244.9, where two glucopyranosyl groups were removed [22]. In the case of calycosin-7-*O*-glucoside, one glucose molecule was eliminated, and the peak generated at *m/z* 284.9 was designated as Q3 [23], while in chlorogenic acid and 3,5-dicaffeoylquinic acid, the *m/z* 162.9 of the caffeoyl group was set as the Q3 peak [24]. The flavonols, kaempferol and quercetin, were produced by the cleavage of the C-ring, and *m/z* 152.9 and 150.9 were set as the Q3 peak, respectively [25]. The Q3 peak of gomisin A was set at *m/z* 341.0, which is the ion generated by the removing the water molecule, CH_2_O, and CO groups from Q1 [26,27].

#### 2.2.2. Validation of the Developed UPLC–MS/MS Analytical Method

Detailed data such as retention time, linear range, regression equations, *r*^2^, LOD, and LOQ values of each compound are tabulated in Table 6. Briefly, the *r*^2^ value of the calibration curve for each compound plotted in the tested concentration range was >0.995, and the concentrations of LOD and LOQ were calculated to be 0.02–1.06 μg/L and 0.05–3.18 μg/L, respectively. The recovery was 86.27–99.62% (RSD < 10%), which was appropriately assessed as ±20% (Table 7). Intra- and inter-day precision based on RSD values were measured to be 0.57–9.09%, and the developed analysis method was found to be suitable at <20% (Table 8).

#### 2.2.3. Simultaneous Determination of the Nine Target Components in 70% Ethanol Extract of Freeze-Dried BPT

The contents of the nine investigated compounds in the 70% ethanol extract of freeze-dried BPT were 0.04−4.70 mg/g (Table 9). Among the herbal medicine components of BPT, hydroxymethylfurfural and mulberroside A (the main compounds *R. glutinosa* and *M. alba*) were detected at the highest levels of 4.70 mg/g and 0.74 mg/g, respectively. These results showed a similar pattern to the results of analysis using HPLC–PDA.

## 3. Materials and Methods

### 3.1. Plant Materials

The six raw herbal medicines (see Table S4) were purchased from Kwangmyungdang Pharmaceutical (Ulsan, Republic of Korea). Prior to use, they were subjected to morphological sensory tests by Dr. Goya Choi, Korea Institute of Oriental Medicine (KIOM, Daejeon, Republic of Korea). Scientific names were verified from the World Folra Online Plant List (www.wfoplantlist.org; 21 November 2023) [28]. Six crude herbs (CA05–1 to CA05–6) were stored in the KM Science Research Division, KIOM.

### 3.2. Chemicals and Reagents

The reference target compounds used in this simultaneous quantification were purchased from specialized natural product manufacturing companies: hydroxymethylfurfural and chlorogenic acid from Merck KGaA (Darmstadt, Germany); mulberroside A from Ensol BioSciences (Daejeon, Republic of Korea); calycosin-7-*O*-glucoside, 3,5-dicaffeoylquinic acid, kaempferol, and gomisin A from Shanghai Sunny Biotech (Shanghai, China); quercetin from ChemFaces Biochemical (Wuhan, China); and schizandrin from Biopurify Phytochemicals (Chengdu, China). Detailed information on the structures of these compounds is given in Table S5 and Figure S2, respectively. For analysis, all solvents (i.e., methanol, acetonitrile, and distilled water) and reagents (i.e., formic acid, trifluoroacetic acid, phosphoric acid, and acetic acid) were either HPLC or LC–MS grade. They were purchased from JT Baker (Phillipsburg, NJ, USA), Merck (Darmstadt, Germany), or Thermo Fisher Scientific (Cleveland, OH, USA).

### 3.3. Preparation of the BPT Sample

Preparation of the BPT sample was conducted at KIOM following previously reported preparation protocols [29,30,31]. Briefly, after mixing the amounts as shown in Table S4 (each at 1500 g; *M. alba* and *R. glutinosa*, each at 500 g; *P. ginseng*, *A. tataricus*, *A. propinquus*, and *S. chinensis*), 50 L of distilled water was added, and the mixture was boiled at 100 °C for 2 h using a COSMOS-660 heating extractor (Kyungseo E&P, Incheon, Republic of Korea). The extract was lyophilized to obtain a powder sample (1600 g, yield 32.0%). The lyophilized sample was stored at −20 °C until it was required for use.

### 3.4. Equipment and Analytical Conditions for HPLC–PDA Simultaneous Quantification

A Prominence LC-20A series HPLC system (Shimadzu, Kyoto, Japan) was used to analyze nine target components from a BPT sample simultaneously. The system comprised two mobile phase delivery units (i.e., pumps), an online degasser, a column oven with forced air circulation, an autosampler with cooling, and a photodiode array detector. These systems were controlled using LC solution software (version 1.24; Shimadzu). The nine targets (i.e., hydroxymethylfurfural, mulberroside A, chlorogenic acid, calycosin-7-*O*-glucoside, 3,5-dicaffeoylquinic acid, quercetin, kaempferol, schizandrin, and gomisin A) were separated, without any other interfering peaks, using a Waters SunFire^TM^ reverse-phase analytical column (250 mm length × 4.6 mm inner diameter, particle size 5 μm; Waters, Milford, MA, USA) and a distilled water–acetonitrile (both containing 0.1% (*v/v*) formic acid) gradient elution condition. Further details of the HPLC analysis conditions are given in Table S1.

### 3.5. Equipment and Analytical Conditions for UPLC–MS/MS Simultaneous Quantification

The simultaneous quantification of nine target compounds in a BPT sample was performed using a UPLC–MS/MS system comprising a Waters Acquity UPLC H-Class PLUS system and a TQ-S micro-MS system (Xevo, Milford, MA, USA). The operation conditions are given in Table S6. Various parameters for UPLC–MS/MS MRM analysis of targets are given in Table 5. These include the ion mode, MRM transition, cone voltage, and collision energy.

### 3.6. Validation of Established Assays in HPLC–PDA and UPLC–MS/MS Systems

Based on guidelines from the International Conference on Harmonization [32], linearity, sensitivity, such as the LOD and LOQ, accuracy, and precision were evaluated to verify the established assays. Briefly, in both methods, the linearity was evaluated by the coefficient of determination (*r*^2^) value in the regression equation for each analyte. In the case of the HPLC–PDA method, the LOD and LOQ concentrations of each compound were calculated using the following equation:

$$LOD=3.3\;\frac{\sigma }{S}\;\mathrm{and}\;LOQ=10\;\frac{\sigma }{S}$$

where *σ* is the standard deviation (SD) of the *y*-intercept, and *S* is the slope of the regression equation.

On the other hand, in the UPLC–MS/MS method, the LOD and LOQ concentrations were calculated using signal-to-noise ratios of 3:1 and 10:1, respectively.

Determination of the recovery was conducted using the standard addition method with three different concentrations (i.e., low, medium, and high) of the target compounds. The recovery parameter was calculated based on the following equation:

$$Recovery\left(\%\right)=\frac{found\;amount}{spiked\;amount}\times 100$$

The intra- and inter-day precisions of the established assays were measured using mixed standard solutions of three different levels (i.e., low, medium, and high) for 1 day and for 3 consecutive days and then verified by RSD values. RSD was calculated using the following equation:

$$RSD\left(\%\right)=\frac{SD}{Mean}\times 100$$

## 4. Conclusions

Herein, use was made of HPLC and UPLC–MS/MS analyses to develop a simultaneous analytical method for a sample with nine target compounds for the quality control of BPT. BPT has been traditionally used for the treatment of respiratory diseases. The analytical methods of the two developed systems were verified by evaluating various parameters, such as linearity, sensitivity (LOD and LOQ), accuracy, and precision. Generally, the UPLC−MS/MS MRM method offered the advantage of enabling multicomponent analysis with high sensitivity in a short analysis time, compared to the HPLC method. The HPLC analysis method is nonetheless widely used and simple to operate, and it is being increasingly used as an analytical method for the quality control of traditional herbal medicines. In this study, results of quantitative analysis from the two methods indicated that hydroxymethylfurfural is the most abundant component in BPT. Based on this knowledge to date and the results gathered, the author believes that the herein developed and validated assays can, in the future, be used to obtain basic data for clinical and efficacy studies.

## Figures and Tables

**Table 1:** Wavelength, linear range, regression equation, coefficient of determination (*r*^2^), limit of detection (LOD), and limit of quantitation (LOQ) values for the simultaneous analysis of the selected nine target compounds in the HPLC–PDA method.

Analyte ^1^	Detected Wavelength (nm)	Linear Range (μg/mL)	Regression Equation ^2^ y=ax+b	*r* ^2^	LOD (μg/mL)	LOQ (μg/mL)
1	280	0.47–30.00	*y* = 84,054.98*x* + 12,165.26	0.9999	0.06	0.17
2	325	0.78–50.00	*y* = 17,148.00*x* + 2048.13	1.0000	0.08	0.26
3	325	0.31–20.00	*y* = 37,034.13*x* + 2053.14	1.0000	0.02	0.05
4	250	0.47–30.00	*y* = 54,712.87*x* + 6199.80	1.0000	0.05	0.16
5	325	0.31–20.00	*y* = 36,855.19*x* + 938.58	1.0000	0.03	0.10
6	370	0.31–20.00	*y* = 36,647.35*x* + 1188.71	1.0000	0.05	0.16
7	365	0.31–20.00	*y* = 43,096.32*x* + 2275.32	1.0000	0.05	0.15
8	250	0.31–20.00	*y* = 20,953.10*x* + 1163.90	1.0000	0.01	0.04
9	250	0.31–20.00	*y* = 19,636.01*x* + 1391.43	1.0000	0.05	0.16

^1^ Hydroxymethylfurfural (1), mulberroside A (2), chlorogenic acid (3), calycosin-7-*O*-glucoside (4), 3,5-dicaffeoylquinic acid (5), quercetin (6), kaempferol (7), schizandrin (8), and gomisin A (9). ^2^ *y*: peak area of compounds; *x*: concentration (μg/mL) of compounds.

**Table 2:** Recovery (%) of the selected nine target compounds in the established HPLC–PDA method.

Analyte ^1^	Spiked Amount (μg/mL)	Found Amount (μg/mL)	Recovery (%)	SD ^2^	RSD ^3^ (%)
1	1.00	0.98	97.62	0.51	0.53
	2.00	2.05	102.63	0.82	0.80
	4.00	4.17	104.15	1.06	1.02
2	4.00	4.09	102.34	1.98	1.93
	10.00	10.17	101.71	1.47	1.44
	20.00	21.28	106.40	0.63	0.59
3	1.00	0.99	99.00	1.77	1.78
	2.00	2.03	101.26	1.49	1.47
	4.00	4.02	100.60	1.16	1.15
4	1.00	1.02	102.13	1.28	1.25
	2.00	2.05	102.55	0.46	0.45
	4.00	4.14	103.49	0.78	0.75
5	1.00	1.00	100.45	0.89	0.89
	2.00	2.05	102.37	1.01	0.99
	4.00	4.05	101.34	0.74	0.73
6	1.00	0.99	99.32	0.77	0.77
	2.00	1.94	96.91	0.42	0.44
	4.00	3.84	95.93	0.32	0.33
7	1.00	0.99	99.07	0.65	0.66
	2.00	2.03	101.62	0.25	0.24
	4.00	4.01	100.24	0.32	0.32
8	1.00	1.01	100.51	1.49	1.48
	2.00	2.04	102.06	0.56	0.55
	4.00	4.02	100.44	0.52	0.52
9	1.00	1.01	101.20	0.70	0.69
	2.00	2.02	101.24	1.37	1.36
	4.00	4.09	102.25	0.44	0.43

^1^ Hydroxymethylfurfural (1), mulberroside A (2), chlorogenic acid (3), calycosin-7-*O*-glucoside (4), 3,5-dicaffeoylquinic acid (5), quercetin (6), kaempferol (7), schizandrin (8), and gomisin A (9). ^2^ SD: standard deviation. ^3^ Relative standard deviation.

**Table 3:** Precision test of the nine target compounds in the established HPLC–PDA method.

Analyte ^1^	Conc. (μg/mL)	Intra-Day (*n* = 5)				Inter-Day (*n* = 5)	
		Observed Conc. (μg/mL)	Precision (RSD, %)	Accuracy (%)	Observed Conc. (μg/mL)	Precision (RSD, %)	Accuracy (%)
1	7.50	7.49	0.72	99.83	7.40	2.53	97.95
	15.00	15.12	0.76	100.83	15.17	1.36	101.14
	30.00	29.51	0.16	98.36	29.67	0.77	98.89
2	12.50	12.59	0.35	100.74	12.30	3.09	98.82
	25.00	25.19	1.55	100.78	25.68	2.38	102.70
	50.00	49.64	0.34	99.27	50.27	1.61	100.54
3	5.00	4.91	0.73	98.16	4.84	3.30	97.13
	10.00	9.96	1.06	99.63	10.09	1.93	100.87
	20.00	19.65	0.22	98.23	19.89	1.34	99.47
4	7.50	7.52	0.69	100.26	7.39	2.71	97.93
	15.00	15.16	1.34	101.09	15.44	2.33	102.94
	30.00	29.90	0.41	99.67	30.24	1.61	100.79
5	5.00	4.94	0.53	98.74	4.86	3.06	97.67
	10.00	9.98	1.33	99.77	10.18	2.42	101.83
	20.00	19.86	0.36	99.29	20.07	1.48	100.35
6	5.00	4.95	0.77	99.08	4.87	3.02	97.73
	10.00	10.02	1.16	100.23	10.17	1.97	101.66
	20.00	19.88	0.32	99.42	20.09	1.36	100.44
7	5.00	4.97	0.67	99.37	4.89	2.75	97.81
	10.00	10.08	1.05	100.76	10.21	1.95	102.11
	20.00	19.94	0.51	99.69	20.15	1.51	100.76
8	5.00	4.99	0.55	99.87	4.91	2.75	98.21
	10.00	10.08	1.18	100.82	10.20	1.92	101.98
	20.00	19.96	0.43	99.81	20.17	1.45	100.85
9	5.00	4.99	0.67	99.87	4.93	2.80	98.53
	10.00	10.09	1.09	100.86	9.58	1.90	95.78
	20.00	19.95	0.42	99.77	18.89	1.40	94.46

^1^ Hydroxymethylfurfural (1), mulberroside A (2), chlorogenic acid (3), calycosin-7-*O*-glucoside (4), 3,5-dicaffeoylquinic acid (5), quercetin (6), kaempferol (7), schizandrin (8), and gomisin A (9).

**Table 4:** Amounts (mg/g) of the nine target compounds in the BPT sample by the established HPLC–PDA assay.

Analyte ^1^	HPLC−PDA Assay		
	Mean (mg/g)	SD × 10^−2^	RSD (%)
1	3.02	1.43	0.48
2	1.81	2.72	1.50
3	0.39	0.39	1.00
4	0.07	0.06	0.88
5	0.11	0.26	2.32
6	0.02	0.02	1.10
7	0.02	0.04	1.84
8	0.10	0.10	0.99
9	0.01	0.01	1.52

^1^ Hydroxymethylfurfural (1), mulberroside A (2), chlorogenic acid (3), calycosin-7-*O*-glucoside (4), 3,5-dicaffeoylquinic acid (5), quercetin (6), kaempferol (7), schizandrin (8), and gomisin A (9).

**Table 5:** Optimized parameters for the UPLC–MS/MS MRM simultaneous analysis of the nine analytes in BPT.

Analyte ^1^	Ion Mode	Molecular Weight	MRM Transition		Cone Voltage (V)	Collision Energy (eV)
			Precursor Ion	Production Ion		
1	+	126.0	126.9	109.0	25	8
2	+	568.2	569.0	244.9	32	16
3	+	354.1	355.0	162.9	36	14
4	+	446.1	447.0	284.9	32	16
5	+	516.1	517.1	162.9	10	22
6	−	302.0	300.8	150.9	52	20
7	+	286.1	286.9	152.9	64	28
8	+	432.2	433.0	415.0	26	8
9	+	416.2	417.1	341.0	44	16

^1^ Hydroxymethylfurfural (1), mulberroside A (2), chlorogenic acid (3), calycosin-7-*O*-glucoside (4), 3,5-dicaffeoylquinic acid (5), quercetin (6), kaempferol (7), schizandrin (8), and gomisin A (9).

**Table 6:** Retention time, the linear range, regression equation, *r*^2^, LOD, and LOQ of the nine compounds by the UPLC–MS/MS MRM analytical method.

Analyte ^1^	Retention Time (min)	Linear Range (μg/L)	Regression Equation ^2^ y=ax+b	*r* ^2^	LOD (μg/L)	LOQ (μg/L)
1	2.20	78.10−1250.00	*y* = 710.12*x* + 34,295.70	0.9983	0.45	1.36
2	3.41	15.60−250.00	*y* = 178.53*x* + 591.23	0.9953	1.06	3.18
3	3.67	62.50−1000.00	*y* = 335.50*x* + 2926.29	0.9993	0.22	0.66
4	5.25	10.90−175.00	*y* = 2309.15*x* + 1839.68	0.9982	0.02	0.05
5	5.93	10.90−175.00	*y* = 52.14*x* + 392.15	0.9952	0.50	1.51
6	7.52	46.90−750.00	*y* = 9.49*x* + 429.68	0.9989	0.92	2.77
7	8.56	62.50−1000.00	*y* = 120.00*x* − 562.67	0.9981	0.69	2.07
8	11.06	23.40−375.00	*y* = 1695.32*x* + 1762.09	0.9990	0.06	0.18
9	11.87	23.40−375.00	*y* = 179.70*x* − 860.67	0.9994	0.26	0.78

^1^ Hydroxymethylfurfural (1), mulberroside A (2), chlorogenic acid (3), calycosin-7-*O*-glucoside (4), 3,5-dicaffeoylquinic acid (5), quercetin (6), kaempferol (7), schizandrin (8), and gomisin A (9). ^2^ *y*: peak area of compounds; *x*: concentration (μg/L) of compounds.

**Table 7:** Recovery (%) of the nine compounds by the developed UPLC–MS/MS MRM analytical method (*n* = 3).

Analyte ^1^	Spiked Amount (μg/L)	Found Amount (μg/L)	Recovery (%)	SD	RSD (%)
1	90.00	79.84	88.71	9.33	2.34
	225.00	195.50	86.89	1.19	0.24
	450.00	406.67	90.37	5.35	0.73
2	15.00	14.16	94.39	2.09	1.92
	37.50	36.96	98.56	2.06	1.51
	75.00	73.53	98.04	3.73	2.1
3	70.00	70.10	100.14	15.94	4.30
	175.00	177.92	101.67	23.75	4.92
	350.00	347.41	99.26	12.82	1.99
4	9.00	7.85	87.20	0.49	1.04
	22.50	19.41	86.27	5.39	9.58
	45.00	39.19	87.08	0.75	1.01
5	11.00	10.96	99.62	2.44	3.71
	27.50	25.56	92.95	1.47	1.91
	55.00	54.42	98.94	1.57	1.44
6	80.00	68.79	85.99	14.45	4.35
	200.00	173.54	86.77	10.57	2.54
	400.00	352.12	88.03	17.49	3.33
7	100.00	87.59	87.59	17.48	4.24
	250.00	215.05	86.02	14.62	2.75
	500.00	440.45	88.09	7.29	1.08
8	30.00	26.55	88.50	0.54	0.34
	75.00	71.91	95.88	0.77	0.35
	150.00	139.34	92.89	0.54	0.19
9	34.00	29.45	86.61	0.67	0.43
	85.00	73.81	86.83	2.66	1.26
	170.00	149.70	88.06	0.43	0.15

^1^ Hydroxymethylfurfural (1), mulberroside A (2), chlorogenic acid (3), calycosin-7-*O*-glucoside (4), 3,5-dicaffeoylquinic acid (5), quercetin (6), kaempferol (7), schizandrin (8), and gomisin A (9).

**Table 8:** Intra- and inter-day precision data of the nine compounds evaluated by the developed UPLC–MS/MS MRM analytical method (*n* = 3).

Analyte ^1^	Conc. (μg/L)	Intra-Day				Inter-Day	
		Observed Conc. (μg/L)	Precision (RSD, %)	Accuracy (%)	Observed Conc. (μg/L)	Precision (RSD, %)	Accuracy (%)
1	156.25	155.12	3.79	99.28	152.89	1.27	97.85
	312.50	338.06	9.09	108.18	338.60	1.42	108.35
	1250.00	1134.75	2.58	90.78	1194.31	4.33	95.54
2	31.25	29.09	9.14	93.08	31.81	7.60	101.78
	62.50	66.16	3.34	105.85	65.40	2.83	104.64
	250.00	237.67	3.98	95.07	241.67	1.62	96.67
3	125.00	131.20	4.02	104.96	126.59	4.77	101.27
	250.00	266.67	4.04	106.67	258.68	3.65	103.47
	1000.00	1068.47	2.17	106.85	1020.08	4.13	102.01
4	21.88	21.53	2.55	98.43	22.16	3.10	101.29
	43.75	42.95	2.73	98.16	45.90	5.61	104.92
	175.00	188.71	1.76	107.84	174.94	6.82	99.97
5	21.88	21.73	2.99	99.33	22.72	3.77	103.85
	43.75	44.01	5.57	100.59	44.63	5.62	102.00
	175.00	177.13	4.18	101.22	172.87	3.21	98.78
6	93.75	92.98	5.89	99.17	96.59	3.67	103.03
	187.50	172.18	7.62	91.83	187.13	6.93	99.80
	750.00	712.02	4.02	94.94	730.76	2.23	97.43
7	125.00	120.87	2.29	96.70	123.54	2.01	98.83
	250.00	239.62	3.93	95.85	252.51	4.54	101.01
	1000.00	972.64	3.05	97.26	973.85	1.05	97.38
8	46.88	48.68	1.60	103.84	48.29	0.91	103.01
	93.75	98.97	0.81	105.57	97.99	1.16	104.52
	375.00	374.83	0.57	99.95	368.78	1.43	98.34
9	46.88	44.55	1.87	95.04	46.12	2.96	98.40
	93.75	95.89	4.07	102.28	94.64	2.51	92.53
	375.00	365.02	2.68	97.34	370.17	1.45	98.71

^1^ Hydroxymethylfurfural (1), mulberroside A (2), chlorogenic acid (3), calycosin-7-*O*-glucoside (4), 3,5-dicaffeoylquinic acid (5), quercetin (6), kaempferol (7), schizandrin (8), and gomisin A (9).

**Table 9:** Amounts (mg/g) of the nine target compounds in a BPT sample, as evaluated by the developed UPLC−MS/MS MRM assays.

Analyte ^1^	UPLC−MS/MS MRM Assay		
	Mean (mg/g)	SD × 10^−1^	RSD (%)
1	4.70	1.88	4.01
2	0.74	0.61	8.26
3	0.30	0.01	2.57
4	0.05	0.01	1.85
5	0.05	0.01	2.00
6	0.04	0.02	5.40
7	0.05	0.04	7.09
8	0.15	0.02	1.05
9	0.17	0.01	0.50

^1^ Hydroxymethylfurfural (1), mulberroside A (2), chlorogenic acid (3), calycosin-7-*O*-glucoside (4), 3,5-dicaffeoylquinic acid (5), quercetin (6), kaempferol (7), schizandrin (8), and gomisin A (9).

## Data Availability

All data in this study can be found in this paper.

## Supplementary Materials

The following supporting information can be downloaded at: https://www.mdpi.com/article/10.3390/molecules29051171/s1, Figure S1: HPLC–PDA chromatograms considered for the selection of target components. A: Mixed 17 standard compounds. B: 70% methanol solution of lyophilized BPT water extract. C: *M. alba* extract. D: *R. glutinosa* extract. E: *P. ginseng* extract. F: *A. tataricus* extract. G: *A. propinquus* extract. H: *S. chinensis* extract. Hydroxymethylfurfural (1), mulberroside A (2), chlorogenic acid (3), rutin (4), calycosin-7-*O*-glucoside (5), isoquercetin (6), 3,4-dicaffeoylquinic acid (7), 3,5-dicaffeoylquinic acid (8), ginsenoside Rg_1_ (9), resveratrol (10), quercetin (11), ginsenoside Rb_1_ (12), kaempferol (13), schizandrin (14), gomisin A (15), astragaloside IV (16), and gomisin N (17); Figure S2: Chemical structures of the nine target components selected for simultaneous analysis in BPT; Figure S3: Comparison of HPLC–PDA chromatograms according to column manufacturer. Standard mixture (A) and BPT sample (B) using the Gemini C_18_ column (Phenomenex, Torrance, CA, USA), standard mixture (C) and BPT sample (D) using the Capcell Pak UG80 C_18_ column (Shiseido, Tokyo, Japan), and standard mixture (E) and BPT sample (F) using the SunFire^TM^ C_18_ column (Waters, Milford, MA, USA). Hydroxymethylfurfural (1), mulberroside A (2), chlorogenic acid (3), rutin (4), calycosin-7-*O*-glucoside (5), isoquercetin (6), 3,4-dicaffeoylquinic acid (7), 3,5-dicaffeoylquinic acid (8), ginsenoside Rg_1_ (9), resveratrol (10), quercetin (11), ginsenoside Rb_1_ (12), kaempferol (13), schizandrin (14), gomisin A (15), astragaloside IV (16), and gomisin N (17); Figure S4: Comparison of HPLC–PDA chromatograms according to the type of acid for the nine selected target compounds. Standard mixture (A) and BPT sample (B) using 0.1% (*v/v*) trifluoroacetic acid, standard mixture (C) and BPT sample (D) using 0.1% (*v/v*) phosphoric acid, standard mixture (E) and BPT sample (F) using 1.0% (*v/v*) acetic acid, and standard mixture (G) and BPT sample (H) using 0.1% (*v/v*) formic acid. Hydroxymethylfurfural (1), mulberroside A (2), chlorogenic acid (3), calycosin-7-*O*-glucoside (4), 3,5-dicaffeoylquinic acid (5), quercetin (6), kaempferol (7), schizandrin (8), and gomisin A (9); Figure S5: Comparison of HPLC–PDA chromatograms according to column temperatures of the nine selected target compounds. Standard mixture (A) and BPT sample (B) at 30 °C, standard mixture (C) and BPT sample (D) at 35 °C, and standard mixture (E) and BPT sample (F) at 40 °C. Hydroxymethylfurfural (1), mulberroside A (2), chlorogenic acid (3), calycosin-7-*O*-glucoside (4), 3,5-dicaffeoylquinic acid (5), quercetin (6), kaempferol (7), schizandrin (8), and gomisin A (9); Figure S6: Extracted ion chromatograms of standard compounds (A) and BPT sample (B) by the UPLC–MS/MS MRM method. Hydroxymethylfurfural (1), mulberroside A (2), chlorogenic acid (3), calycosin-7-*O*-glucoside (4), 3,5-dicaffeoylquinic acid (5), quercetin (6), kaempferol (7), schizandrin (8), and gomisin A (9); Figure S7: Fragmentation of precursor ion (Q1) and product ion (Q3) peaks for each target compound; Table S1: Analytical conditions for the simultaneous analysis of the nine target components in a BPT sample by HPLC–PDA; Table S2: System suitability for the simultaneous analysis of the nine target components by HPLC–PDA; Table S3: Repeatability of retention time and peak area of the nine targets by HPLC (*n* = 6); Table S4: Composition of and information on Bopyeo-tang; Table S5: Information on the nine reference standard compounds; Table S6: UPLC–MS/MS MRM conditions for the simultaneous analysis of nine target components in BPT.
