# Supplementary material for: Simultaneous Quantification of Nine Target Compounds in Traditional Korean Medicine, Bopyeo-Tang, Using High-Performance Liquid Chromatography–Photodiode Array Detector and Ultra-Performance Liquid Chromatography–Tandem Mass Spectrometry
Source: Molecules. 2024 Mar 6;29(5):1171. doi: 10.3390/molecules29051171 (PMC10934735; doi:10.3390/molecules29051171)
Supplement: Supplementary file 1 [file molecules-29-01171-s001.zip › molecules-2883041-supplementary.pdf]

Table S1

Analytical conditions for simultaneous analysis of the nine target components in a BPT sample by HPLC–PDA.

Chromatographic parameter			
Column	SunFire™ C_{18} reverse phase column (250 mm × 4.6 mm, 5 μm)		
Detector	PDA (250, 280, 325, 365, and 370 nm)		
Flow rate	1.0 mL/min		
Injection volume	10.0 μL		
Column temperature	30.0 °C		
Mobile phase	A: 0.1% (v/v) aqueous formic acid<br>B: 0.1% (v/v) formic acid in acetonitrile		
	Time (min)	A (%)	B (%)
Gradient elution	0	95	5
	40	40	60
	50	5	95
	55	5	95
	60	95	5
	70	95	5

PDA; photo-diode array

Table S2

System suitability for simultaneous analysis of the nine target components by HPLC–PDA.

Analyte	*k′*	*α*	*N*	*Rs*	*S*
1	1.24	1.76	36031.97	13.02	1.14
2	2.18	1.32	111828.31	10.70	1.08
3	2.88	1.32	160174.59	10.70	1.20
4	4.65	1.28	428036.39	20.79	1.13
5	5.98	1.28	473439.73	20.79	1.19
6	8.19	1.18	742217.85	20.24	1.16
7	9.69	1.18	911649.05	20.24	1.13
8	12.85	1.09	1028117.50	12.13	1.04
9	13.98	1.09	1201612.01	12.13	1.05

The analytes were hydroxymethylfurfural (1), mulberroside A (2), chlorogenic acid (3), calycosin 7-*O*-glucoside (4), 3,5-dicaffeoylquinic acid (5), quercetin (6), kaempferol (7), schizandrin (8), and gomisin A (9). *k′*; retention factor, *α*; separation factor, *N*; theoretical plate number, *Rs*; resolution, and *S*; symmetry factor

Table S3

Repeatability of retention time and peak area of the nine targets by HPLC (n = 6).

Analyte[1]	Retention time (min)			Peak area (mAU)		
	Mean	SD × 10^{-1}	RSD (%)	Mean	SD	RSD (%)
1	6.36	0.06	0.10	860115.83	4940.92	0.57
2	9.02	0.07	0.07	663327.67	3546.44	0.53
3	11.00	0.06	0.05	702371.17	3837.64	0.55
4	16.03	0.02	0.01	500858.67	3162.93	0.63
5	19.78	0.02	0.01	728009.17	4459.86	0.61
6	26.06	0.03	0.01	719127.33	4588.76	0.64
7	30.31	0.04	0.01	868995.83	5782.18	0.67
8	39.29	0.03	0.01	1067350.67	6562.64	0.61
9	42.48	0.03	0.01	978360.33	5843.46	0.60

[1] Hydroxymethylfurfural (1), mulberroside A (2), chlorogenic acid (3), calycosin 7-*O*-glucoside (4), 3,5-dicaffeoylquinic acid (5), quercetin (6), kaempferol (7), schizandrin (8), and gomisin A (9).

Table S4

Composition of and information on Bopyeo-tang.

Herbal medicine	Scientific name	Family	Using part	Amount (mg)	Ratio (%)
Mori Radicis Cortex	*Morus alba* L.	Moraceae	Root bark	1500.00	30.00
Rehmanniae Radix Preparata	*Rehmannia glutinosa* (Gaertn.) DC.	Plantaginaceae	Root	1500.00	30.00
Ginseng Radix	*Panax ginseng* C.A.Mey.	Araliaceae	Root	500.00	10.00
Asteris Radix et Rhizoma	*Aster tataricus* L.f.	Compositae	Root and rhizome	500.00	10.00
Astragali Radix	*Astragalus propinquus* Schischkin	Lequminosae	Root	500.00	10.00
Schisandrae Fructus	*Schisandra chinensis* (Turcz.) Baill.	Schisandraceae	Fruit	500.00	10.00
			Total	5000.0	100.00

Table S5

Information on the nine reference standard compounds.

Analyte[1]	Purity (%)	Molecular formula	CAS No.	PubChem CID	Catalog No.	Maker
1	≥ 99.0	$C_6H_6O_3$	67-47-0	237332	W501808	Merck KGaA
2	98.1	$C_{26}H_{32}O_{14}$	102841-42-9	6443484	ES030-A	EnsolBioSciences
3	99.7	$C_{16}H_{18}O_9$	327-97-9	1794427	PHL89175	Merck KGaA
4	99.4	$C_{22}H_{22}O_{10}$	20633-67-4	5318267	DR10682	Shanghai Sunny Biotech
5	98.2	$C_{25}H_{24}O_{12}$	2450-53-5	6474310	DR11570	Shanghai Sunny Biotech
6	99.2	$C_{15}H_{10}O_7$	117-39-5	5280343	CFN99272	Wuhan ChemFaces Biochemical
7	≥ 98.0	$C_{15}H_{10}O_6$	520-18-3	5280863	DR10770	Shanghai Sunny Biotech
8	99.3	$C_{24}H_{32}O_7$	7432-28-2	23915	BP1265	Biopurify Phytochemicals
9	99.9	$C_{23}H_{28}O_7$	58546-54-6	634470	DR100561	Shanghai Sunny Biotech

[1] Hydroxymethylfurfural (1), mulberroside A (2), chlorogenic acid (3), calycosin 7-*O*-glucoside (4), 3,5-dicaffeoylquinic acid (5), quercetin (6), kaempferol (7), schizandrin (8), and gomisin A (9).

Table S6

UPLC–MS/MS MRM conditions for simultaneous analysis of nine target components in BPT.

**UPLC conditions**		**MS conditions**	
UPLC system	Acquity UPLC H-Class PLUS	MS system	Xevo TQ-S micro
Column	Acquity UPLC BEH C_{18} column (2.1 mm × 100 mm, 1.7 μm)	MS software	MassLynx v4.2
Column temp.	40 °C	Ion source	ESI^+ or ESI^-
Sample temp.	5 °C	Acquisition mode	MRM
Injection volume	2.0 μL	Capillary voltage	3.3 kV
Flow rate	0.3 mL/min	Cone gas flow	80 L/h
Mobile phase A	0.1% (v/v) acetic acid in distilled water	Desolvation gas flow	600 L/h
Mobile phase B	0.1% (v/v) acetic acid in distilled Acetonitrile	Desolvation temp.	300 °C
Gradient		Source temp.	150 °C

Gradient:

Time (min)	A (%)	B (%)
Initial	95	5
14.43	40	60
14.29	5	95
15.71	5	95
17.14	95	5
20.00	95	5

ESI; electrospray ionization, MRM; multiple reaction monitoring

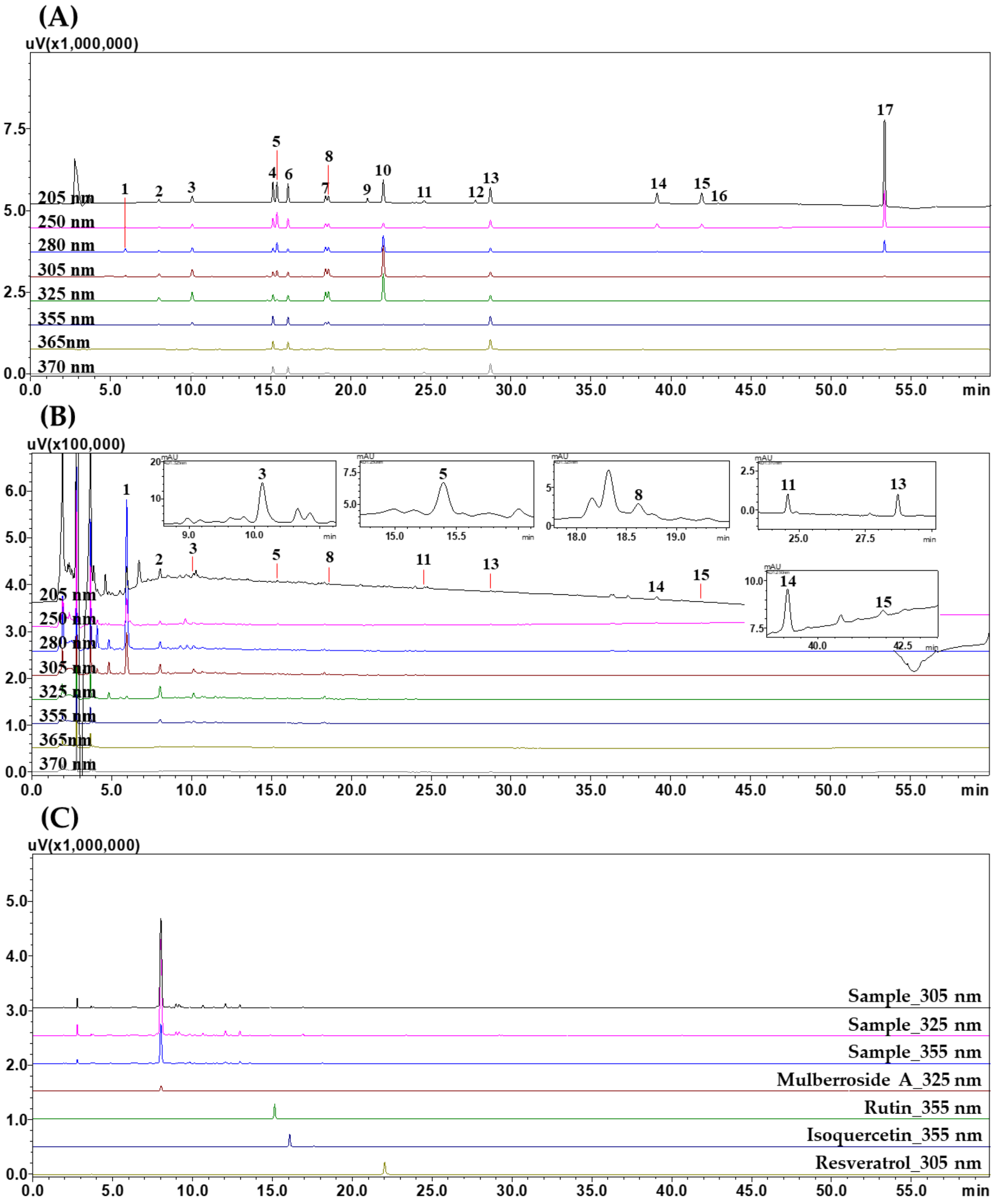

(A)
uV(x1,000,000)
205 nm
250 nm
280 nm
305 nm
325 nm
355 nm
365nm
370 nm
min
(B)
uV(x100,000)
205 nm
250 nm
280 nm
305 nm
325 nm
355 nm
365nm
370 nm
mAU
min
(C)
uV(x1,000,000)
Sample_305 nm
Sample_325 nm
Sample_355 nm
Mulberroside A_325 nm
Rutin_355 nm
Isoquercetin_355 nm
Resveratrol_305 nm
min

(D)

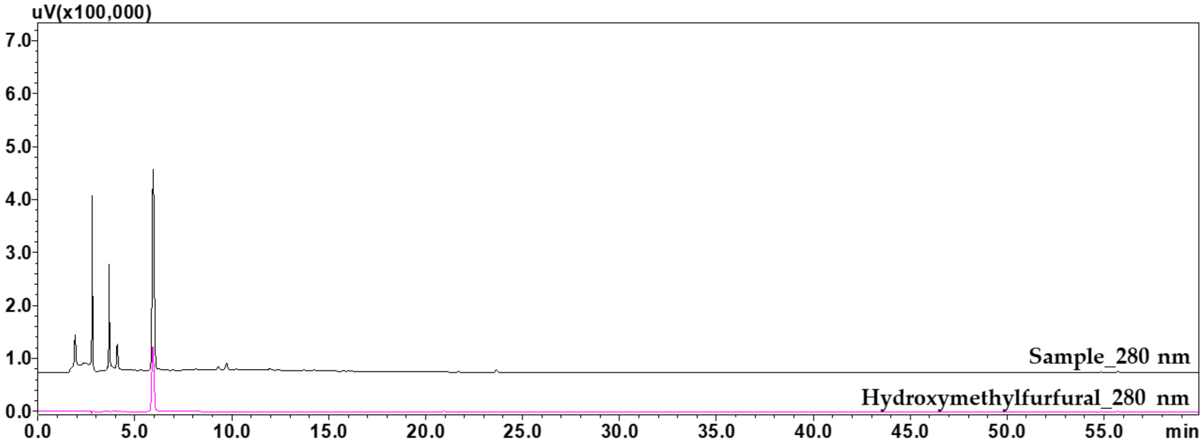

uV(x100,000)
7.0
6.0
5.0
4.0
3.0
2.0
1.0
0.0
Sample_280 nm
Hydroxymethylfurfural_280 nm
0.0
5.0
10.0
15.0
20.0
25.0
30.0
35.0
40.0
45.0
50.0
55.0
min

(E)

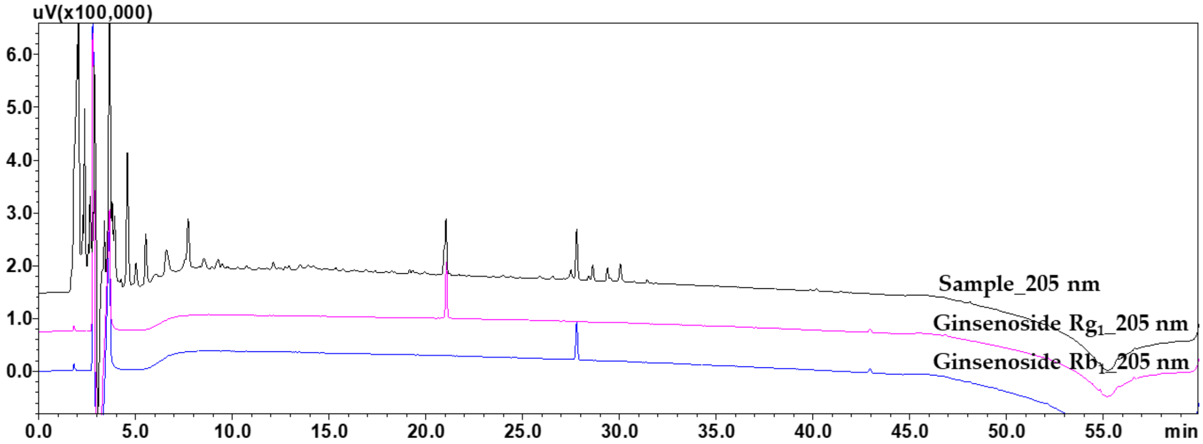

uV(x100,000)
6.0
5.0
4.0
3.0
2.0
1.0
0.0
Sample_205 nm
Ginsenoside Rg1_205 nm
Ginsenoside Rb1_205 nm
0.0
5.0
10.0
15.0
20.0
25.0
30.0
35.0
40.0
45.0
50.0
55.0
min

(F)

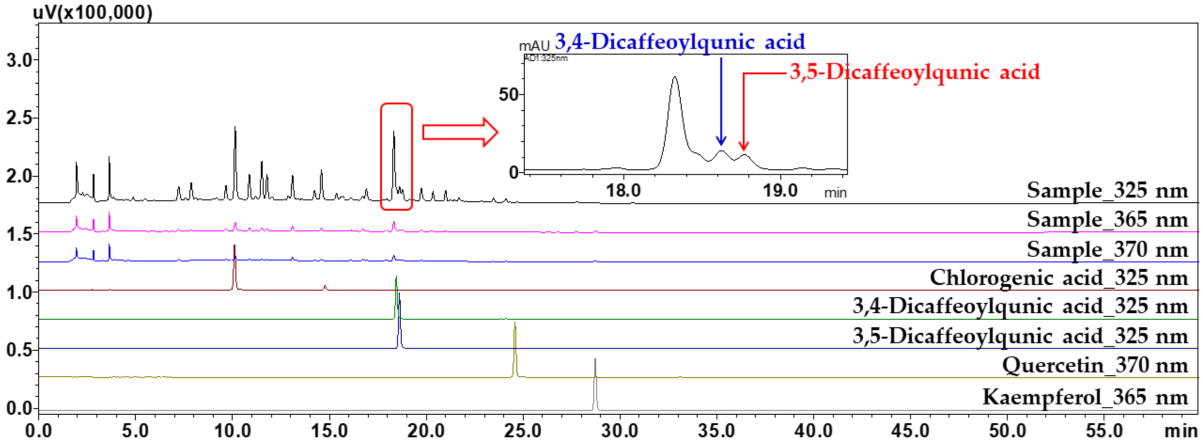

uV(x100,000)
3.0
2.5
2.0
1.5
1.0
0.5
0.0
mAU
3,4-Dicaffeoylqunic acid
3,5-Dicaffeoylqunic acid
50
0
18.0
19.0
min
Sample_325 nm
Sample_365 nm
Sample_370 nm
Chlorogenic acid_325 nm
3,4-Dicaffeoylqunic acid_325 nm
3,5-Dicaffeoylqunic acid_325 nm
Quercetin_370 nm
Kaempferol_365 nm
0.0
5.0
10.0
15.0
20.0
25.0
30.0
35.0
40.0
45.0
50.0
55.0
min

(G)

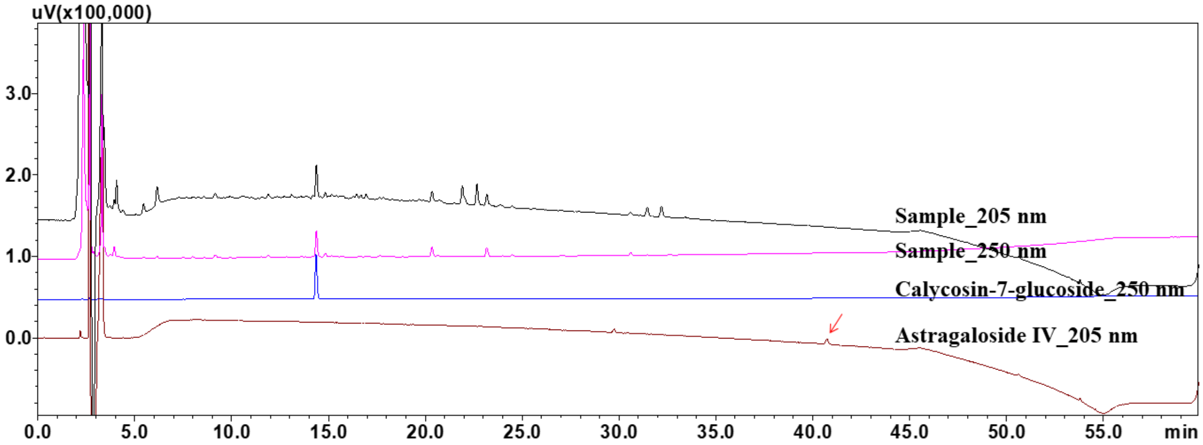

(H)

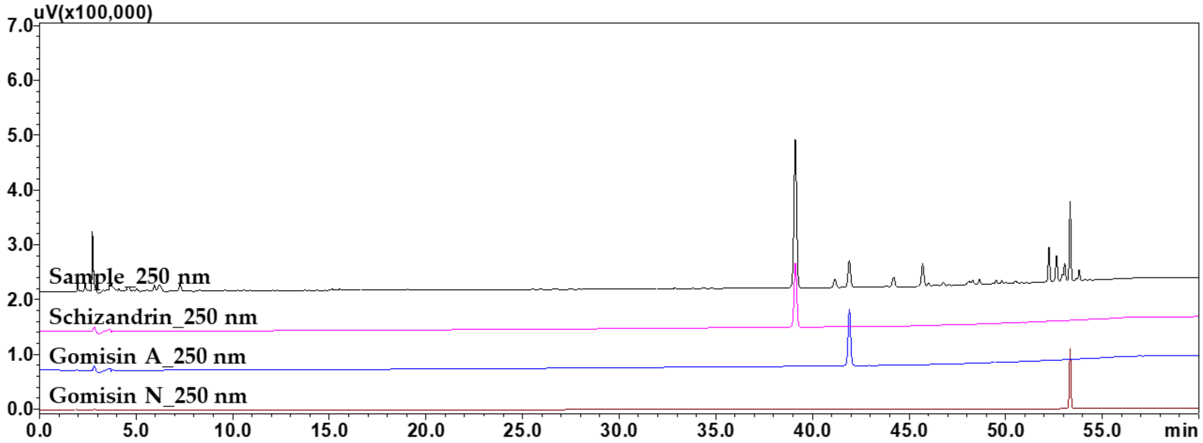

Figure S1. HPLC–PDA chromatograms considered for the selection of target components. A; Mixed 17 standard compounds. B; 70% methanol solution of lyophilized BPT water extract. C; *M. alba* extract. D; *R. glutinosa* extract. E; *P. ginseng* extract. F; *A. tataricus* extract. G; *A. propinquus* extract. H; *S. chinensis* extract. Hydroxymethylfurfural (1), mulberroside A (2), chlorogenic acid (3), rutin (4), calycosin-7-*O*-glucoside (5), isoquercetin (6), 3,4-dicaffeoylquinic acid (7), 3,5-dicaffeoylquinic acid (8), ginsenoside Rg_1 (9), resveratrol (10), quercetin (11), ginsenoside Rb_1 (12), kaempferol (13), schizandrin (14), gomisin A (15), astragaloside IV (16), and gomisin N (17).

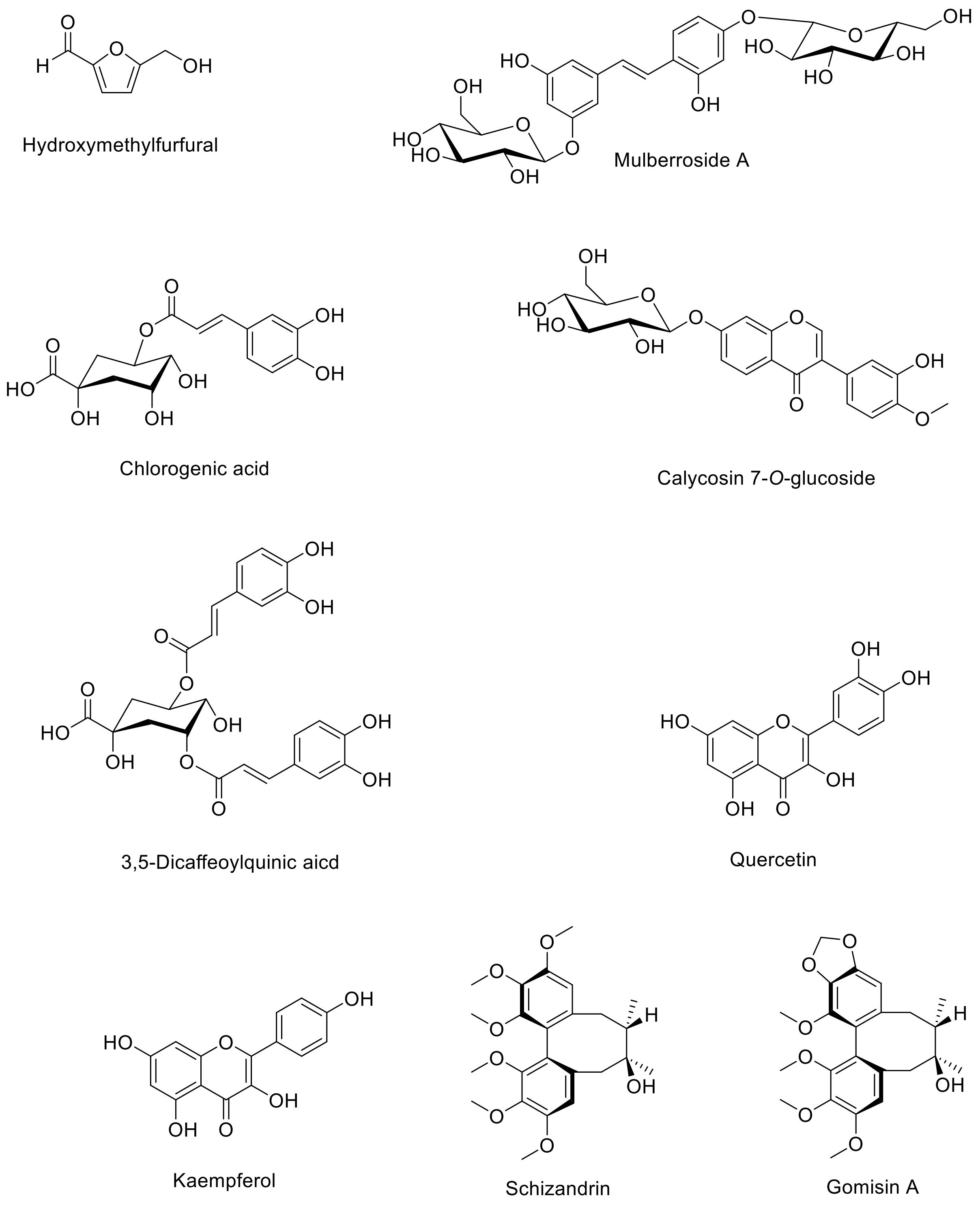

Figure S2. Chemical structures of the nine target components selected for simultaneous analysis in BPT.

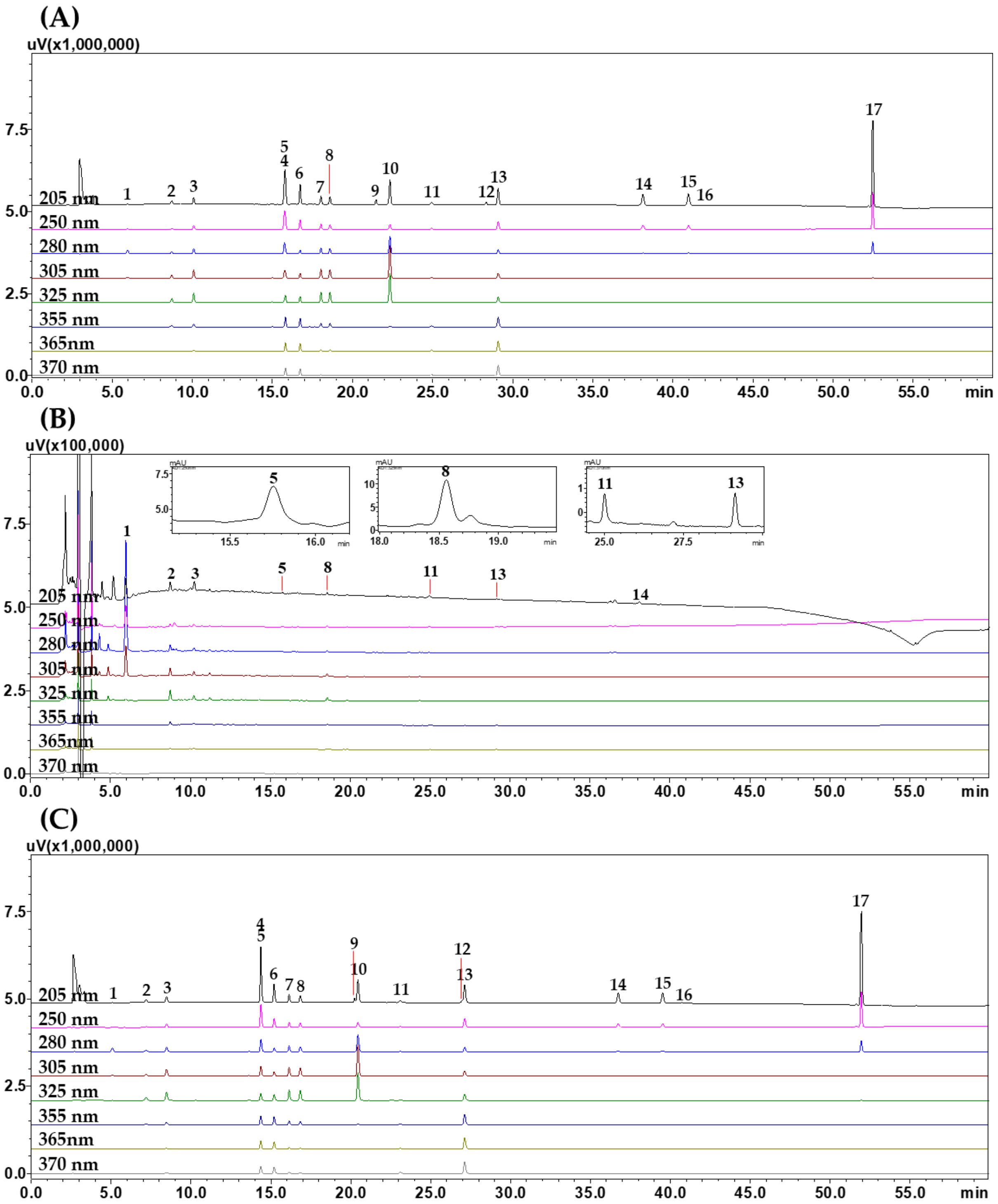
(A)
uV(x1,000,000)
205 nm
250 nm
280 nm
305 nm
325 nm
355 nm
365nm
370 nm
min
(B)
uV(x100,000)
mAU
205 nm
250 nm
280 nm
305 nm
325 nm
355 nm
365nm
370 nm
min
(C)
uV(x1,000,000)
205 nm
250 nm
280 nm
305 nm
325 nm
355 nm
365nm
370 nm
min

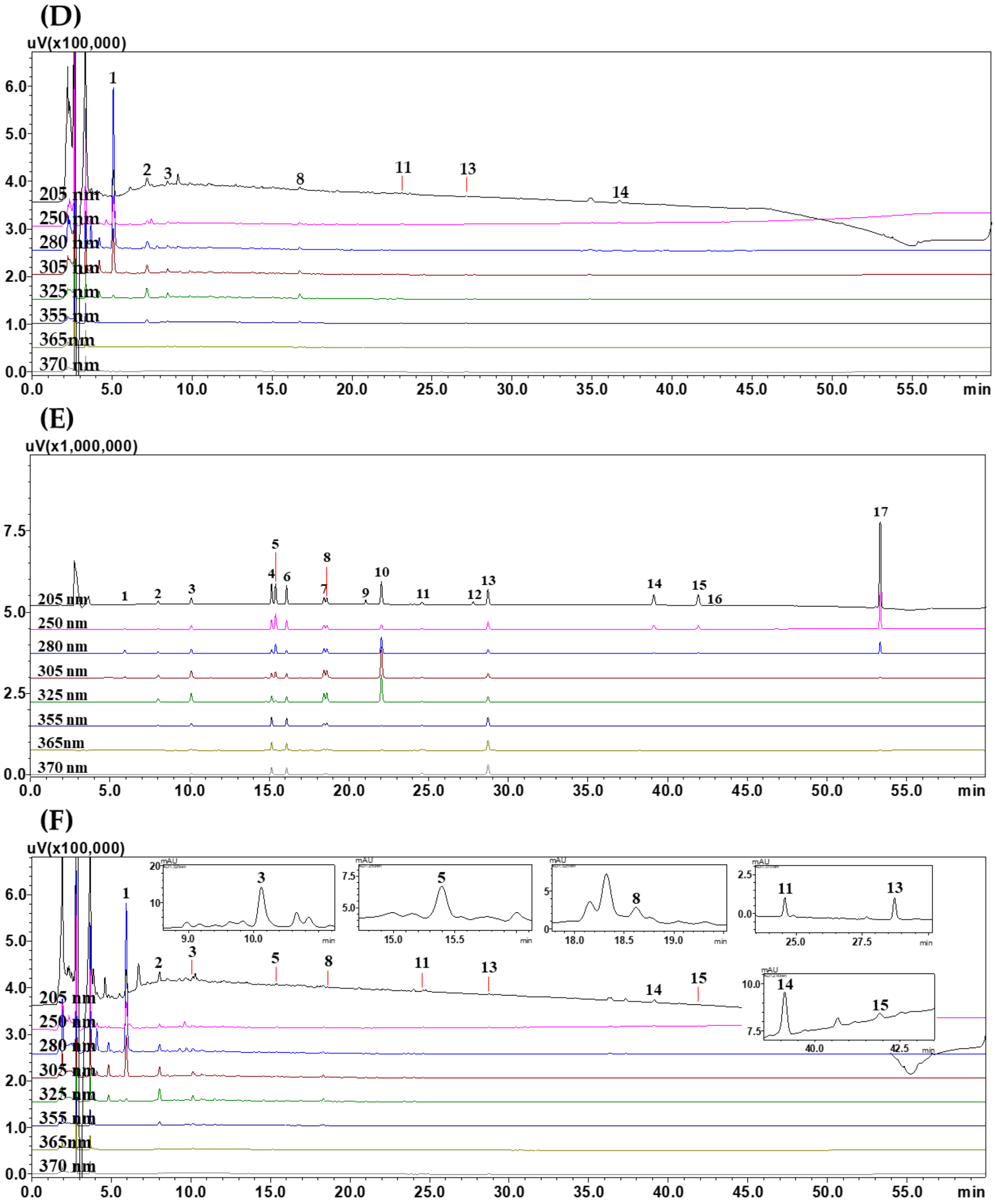

Figure S3. Comparison of HPLC–PDA chromatograms according to column manufacturer. Standard mixture (A) and BPT sample (B) using Gemini C_{18} column (Phenomenex, Torrance, CA, USA), standard mixture (C) and BPT sample (D) using Capcell Pak UG80 C_{18} column (Shiseido, Tokyo, Japan), and standard mixture (E) and BPT sample (F) using SunFire™ C_{18} column (Waters, Milford, MA, USA). Hydroxymethylfurfural (1), mulberroside A (2), chlorogenic acid (3), rutin (4), calycosin-7-*O*-glucoside (5), isoquercetin (6), 3,4-dicaffeoylquinic acid (7), 3,5-dicaffeoylquinic acid (8), ginsenoside Rg_1 (9), resveratrol (10), quercetin (11), ginsenoside Rb_1 (12), kaempferol (13), schizandrin (14), gomisin A (15), astragaloside IV (16), and gomisin N (17).

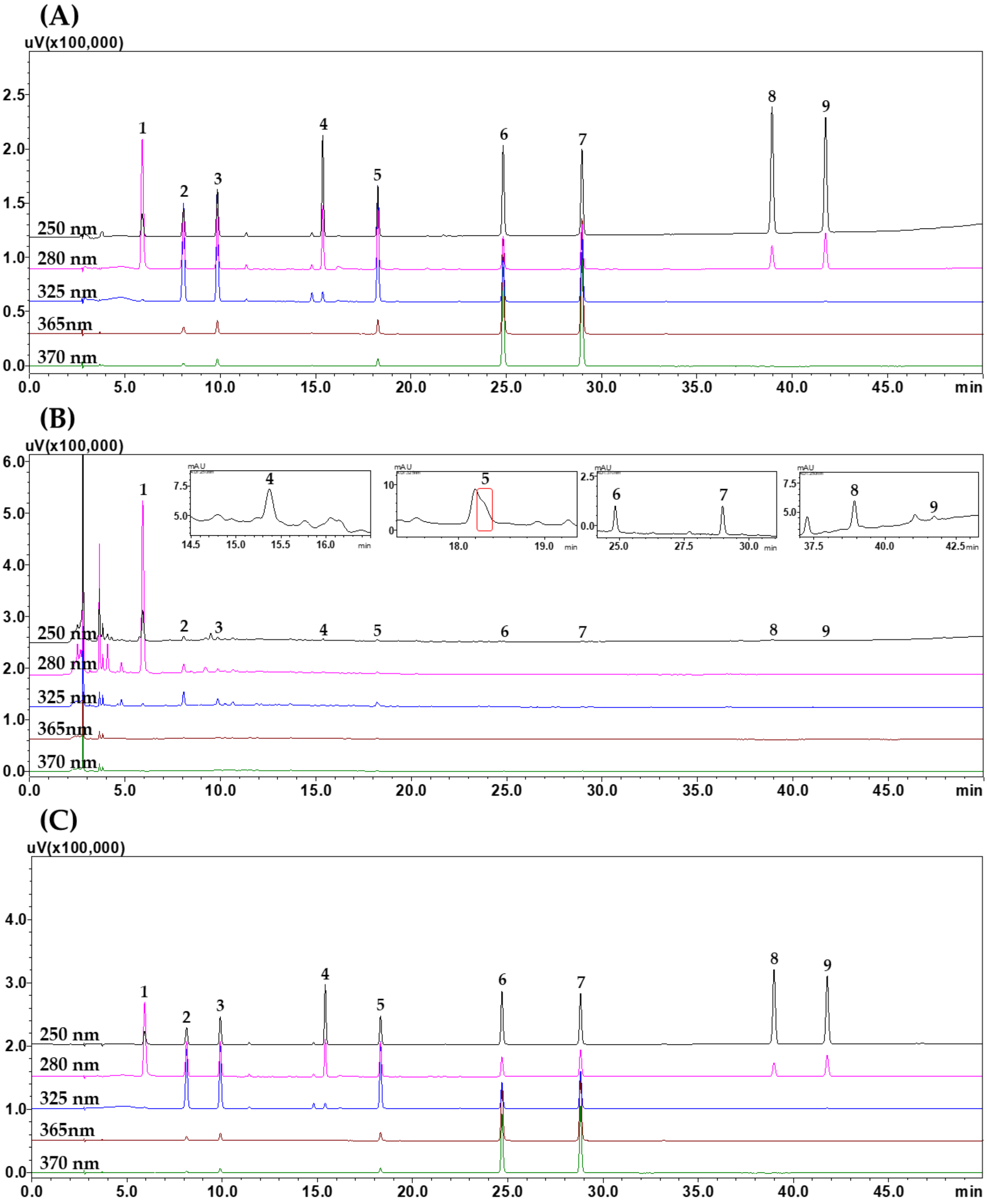
(A)
uV(x100,000)
250 nm
280 nm
325 nm
365nm
370 nm
min
(B)
uV(x100,000)
250 nm
280 nm
325 nm
365nm
370 nm
mAU
min
(C)
uV(x100,000)
250 nm
280 nm
325 nm
365nm
370 nm
min

(D)

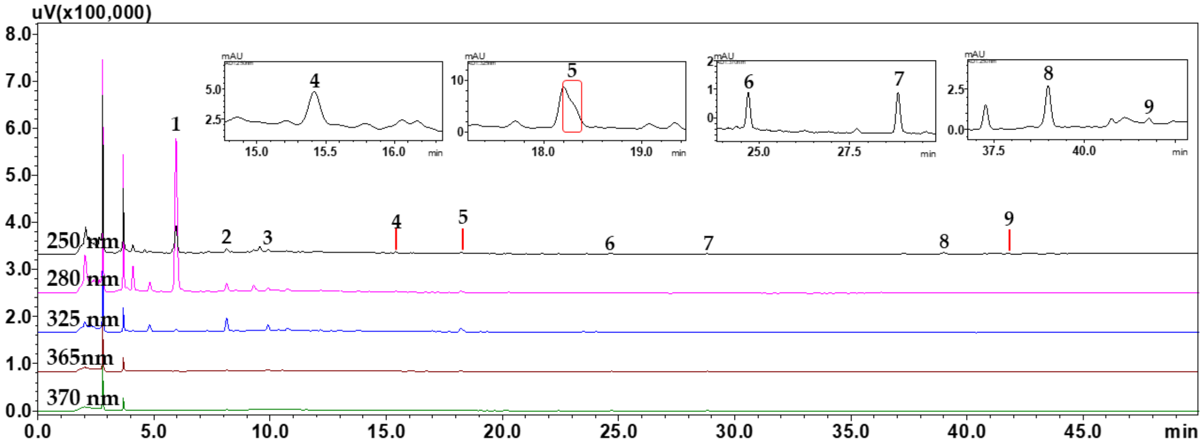
uV(x100,000)
250 nm
280 nm
325 nm
365nm
370 nm
1 2 3 4 5 6 7 8 9
mAU
min

(E)

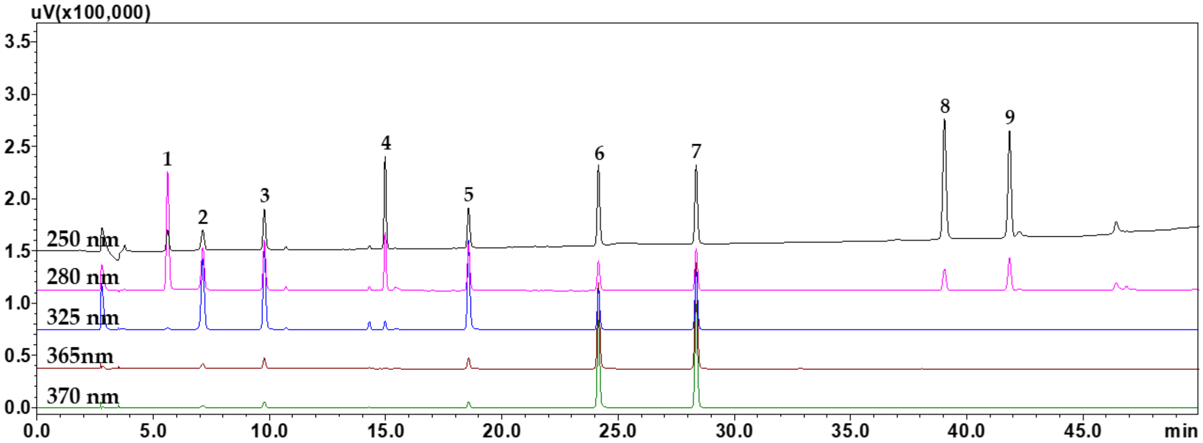
uV(x100,000)
250 nm
280 nm
325 nm
365nm
370 nm
1 2 3 4 5 6 7 8 9
min

(F)

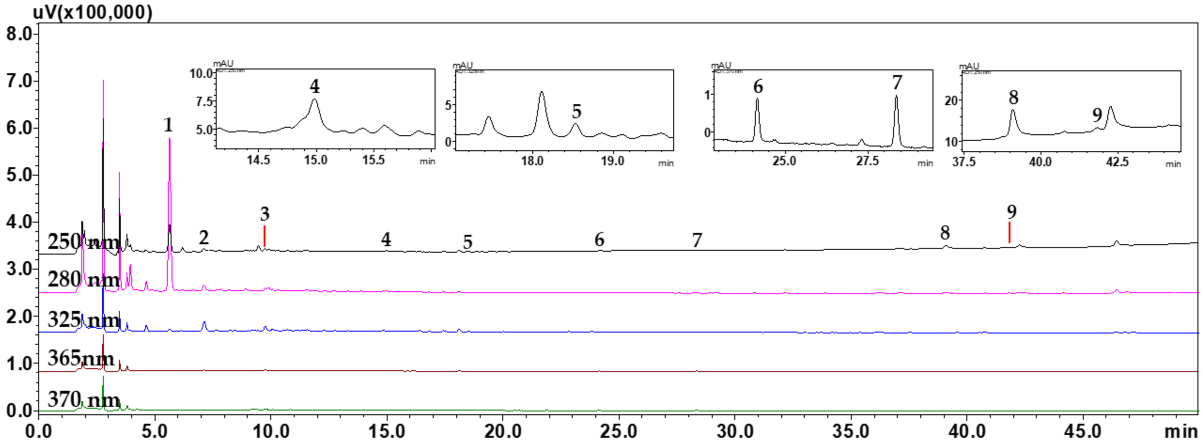
uV(x100,000)
250 nm
280 nm
325 nm
365nm
370 nm
1 2 3 4 5 6 7 8 9
mAU
min

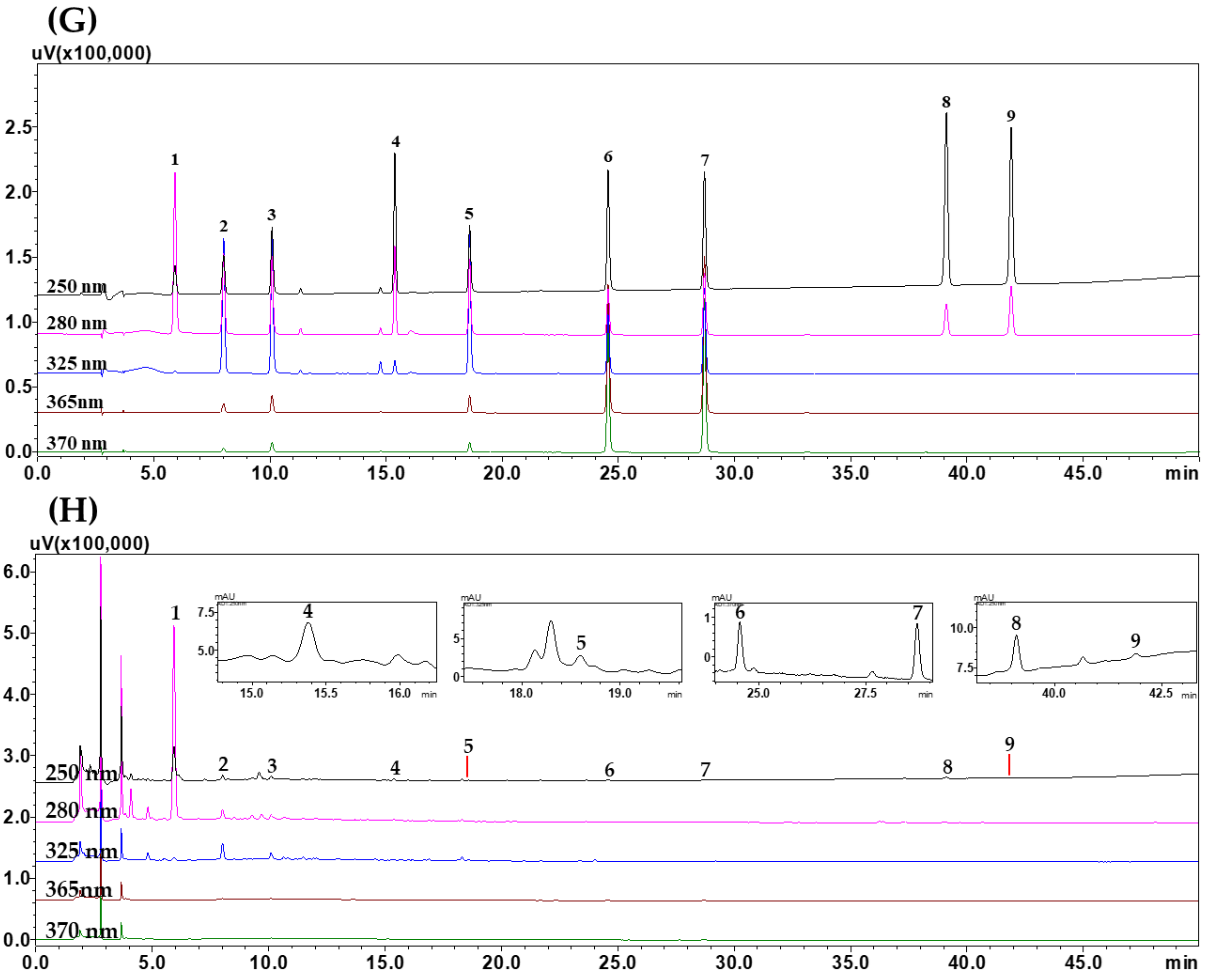

Figure S4. Comparison of HPLC–PDA chromatograms according to the type of acid for the nine selected target compounds. Standard mixture (A) and BPT sample (B) using 0.1% (v/v) trifluoroacetic acid, standard mixture (C) and BPT sample (D) using 0.1% (v/v) phosphoric acid, standard mixture (E) and BPT sample (F) using 1.0% (v/v) acetic acid, and standard mixture (G) and BPT sample (H) using 0.1% (v/v) formic acid. Hydroxymethylfurfural (1), mulberroside A (2), chlorogenic acid (3), calycosin-7-*O*-glucoside (4), 3,5-dicaffeoylquinic acid (5), quercetin (6), kaempferol (7), schizandrin (8), and gomisin A (9).

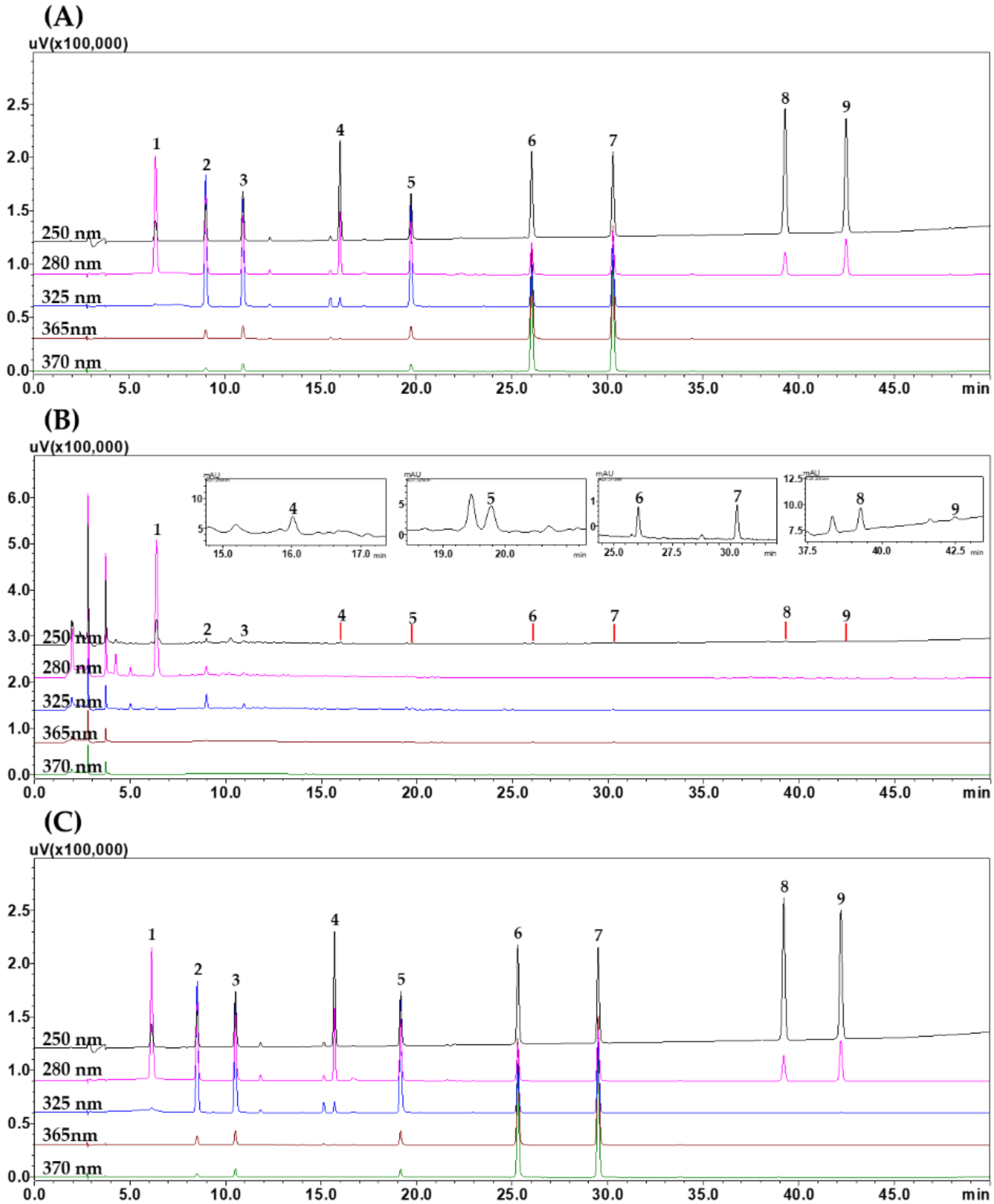
(A)
uV(x100,000)
2.5
2.0
1.5
1.0
0.5
0.0
250 nm
280 nm
325 nm
365nm
370 nm
1
2
3
4
5
6
7
8
9
0.0
5.0
10.0
15.0
20.0
25.0
30.0
35.0
40.0
45.0
min
(B)
uV(x100,000)
6.0
5.0
4.0
3.0
2.0
1.0
0.0
250 nm
280 nm
325 nm
365nm
370 nm
mAU
1
2
3
4
5
6
7
8
9
(C)
uV(x100,000)
2.5
2.0
1.5
1.0
0.5
0.0
250 nm
280 nm
325 nm
365nm
370 nm
1
2
3
4
5
6
7
8
9
min

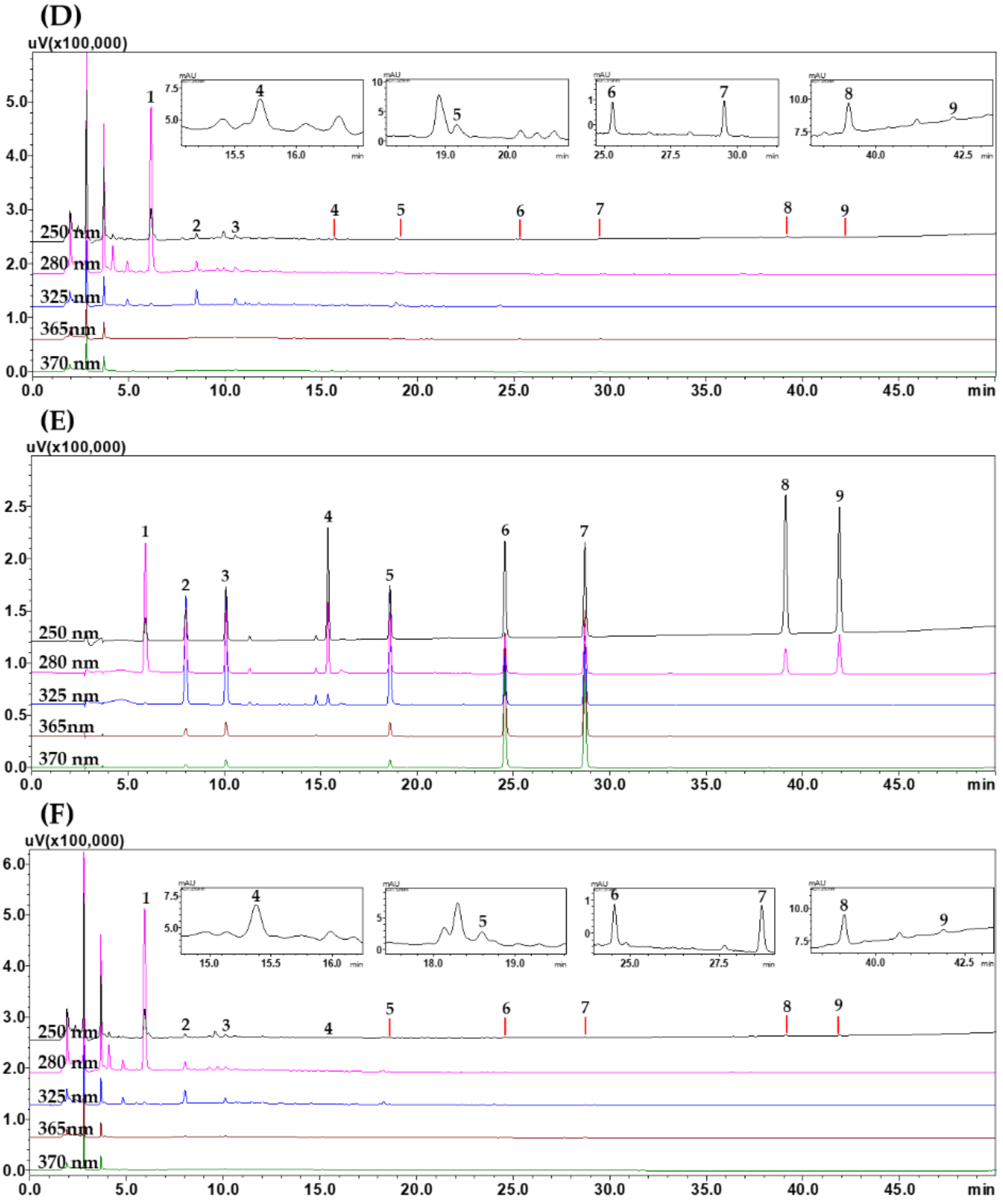

Figure S5. Comparison of HPLC–PDA chromatograms according to column temperatures of the nine selected target compounds. Standard mixture (A) and BPT sample (B) at 30 °C, standard mixture (C) and BPT sample (D) at 35 °C, and standard mixture (E) and BPT sample (F) at 40 °C. Hydroxymethylfurfural (1), mulberroside A (2), chlorogenic acid (3), calycosin-7-*O*-glucoside (4), 3,5-dicaffeoylquinic acid (5), quercetin (6), kaempferol (7), schizandrin (8), and gomisin A (9).

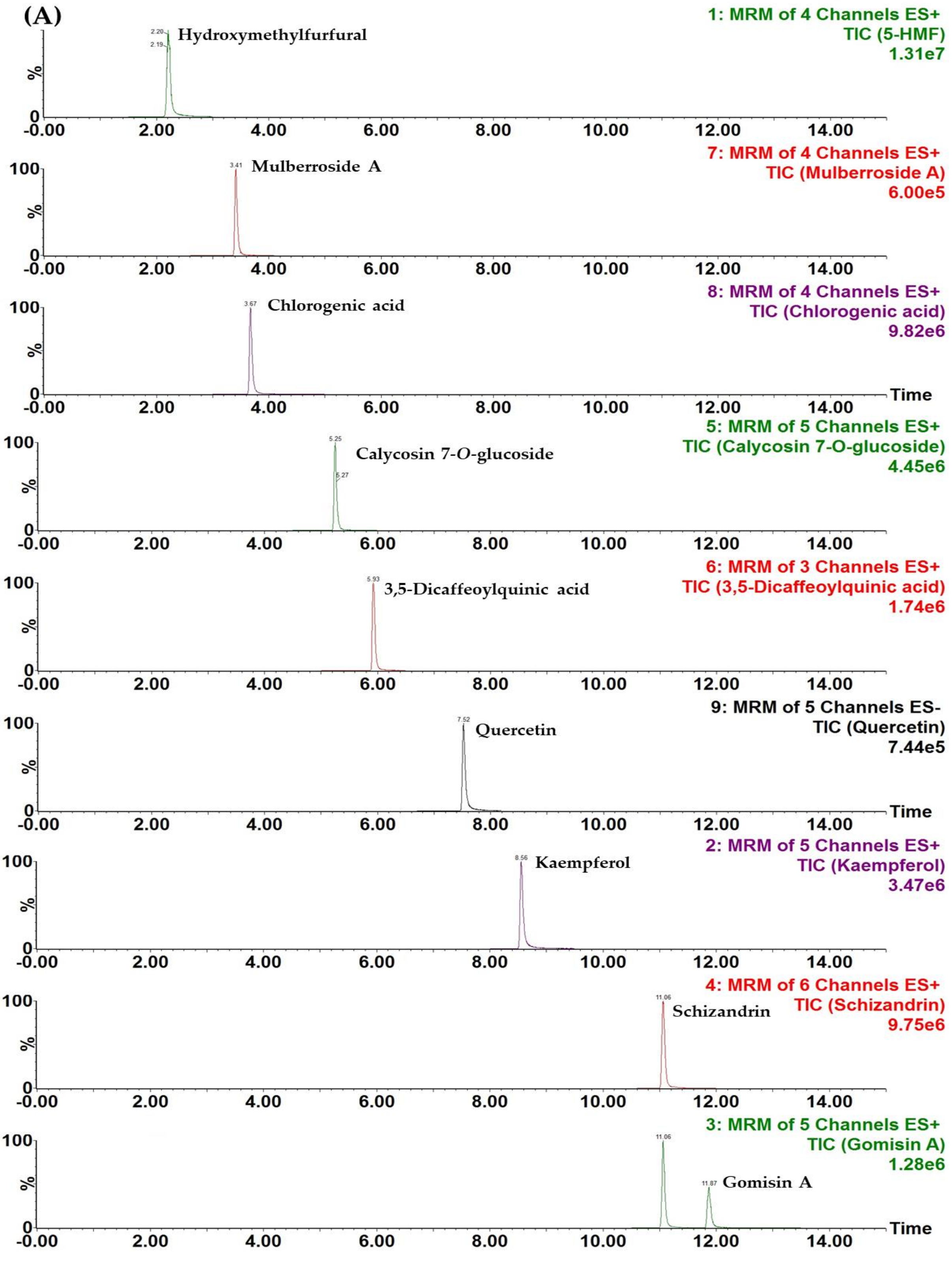
(A)
1: MRM of 4 Channels ES+
TIC (5-HMF)
1.31e7
Hydroxymethylfurfural
7: MRM of 4 Channels ES+
TIC (Mulberroside A)
6.00e5
Mulberroside A
8: MRM of 4 Channels ES+
TIC (Chlorogenic acid)
9.82e6
Chlorogenic acid
5: MRM of 5 Channels ES+
TIC (Calycosin 7-O-glucoside)
4.45e6
Calycosin 7-O-glucoside
6: MRM of 3 Channels ES+
TIC (3,5-Dicaffeoylquinic acid)
1.74e6
3,5-Dicaffeoylquinic acid
9: MRM of 5 Channels ES-
TIC (Quercetin)
7.44e5
Quercetin
2: MRM of 5 Channels ES+
TIC (Kaempferol)
3.47e6
Kaempferol
4: MRM of 6 Channels ES+
TIC (Schizandrin)
9.75e6
Schizandrin
3: MRM of 5 Channels ES+
TIC (Gomisin A)
1.28e6
Gomisin A
Time

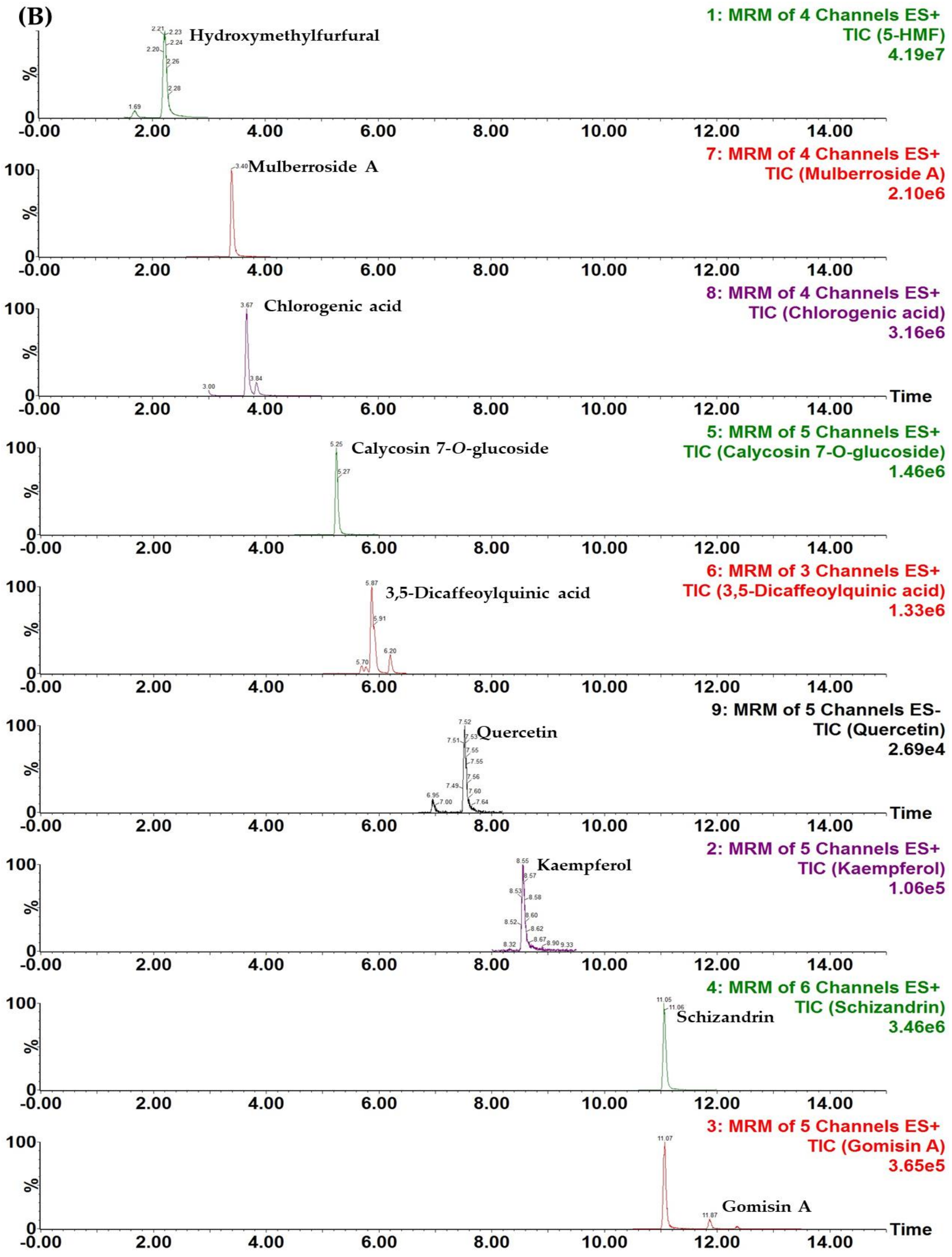

Figure S6. Extracted ion chromatograms of standard compounds (A) and BPT sample (B) by UPLC–MS/MS MRM method. Hydroxymethylfurfural (1), mulberroside A (2), chlorogenic acid (3), calycosin-7-*O*-glucoside (4), 3,5-dicaffeoylquinic acid (5), quercetin (6), kaempferol (7), schizandrin (8), and gomisin A (9).

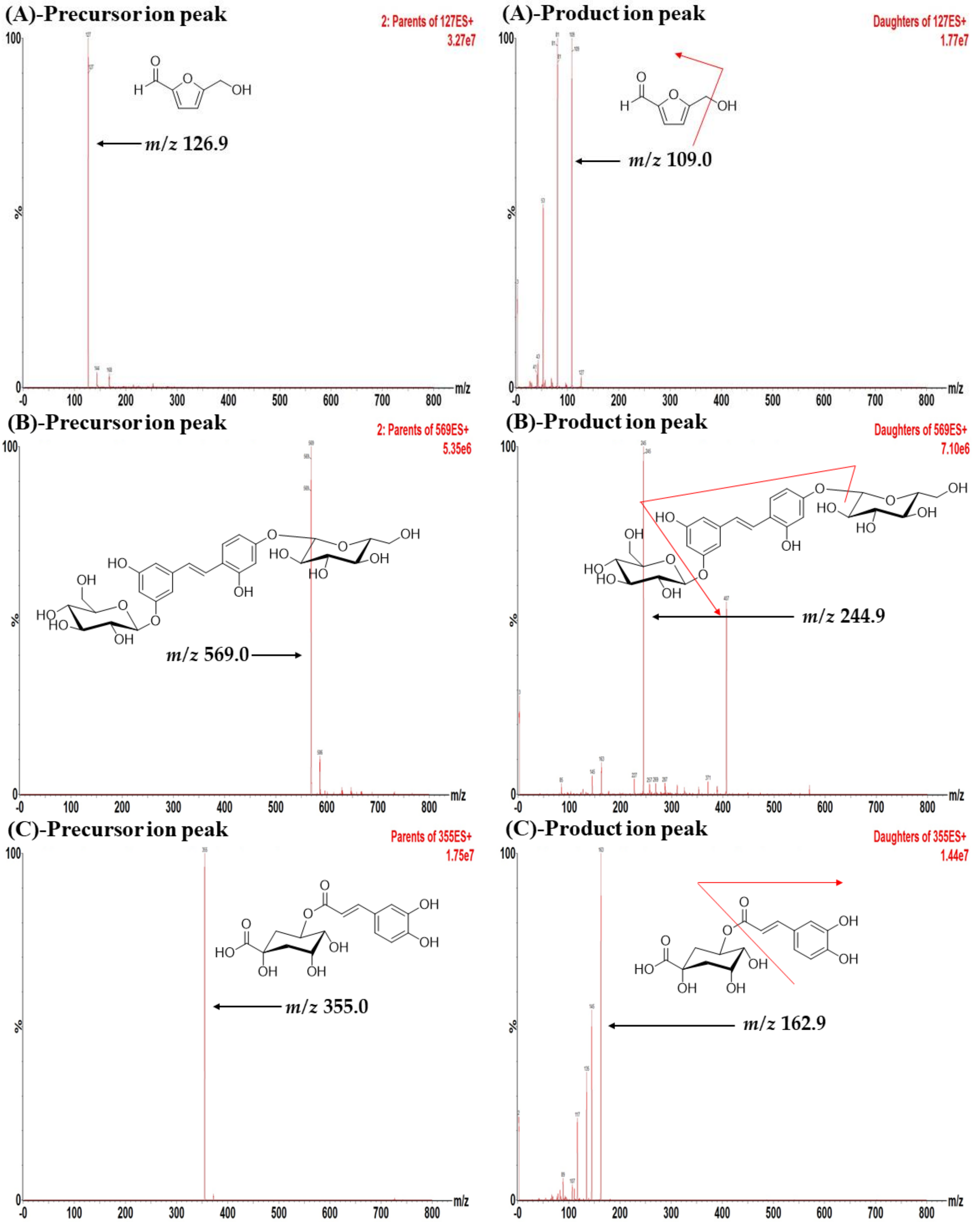

(A)-Precursor ion peak
2: Parents of 127ES+
3.27e7
m/z 126.9
(A)-Product ion peak
Daughters of 127ES+
1.77e7
m/z 109.0
(B)-Precursor ion peak
2: Parents of 569ES+
5.35e6
m/z 569.0
(B)-Product ion peak
Daughters of 569ES+
7.10e6
m/z 244.9
(C)-Precursor ion peak
Parents of 355ES+
1.75e7
m/z 355.0
(C)-Product ion peak
Daughters of 355ES+
1.44e7
m/z 162.9
%
m/z

(D)-Precursor ion peak

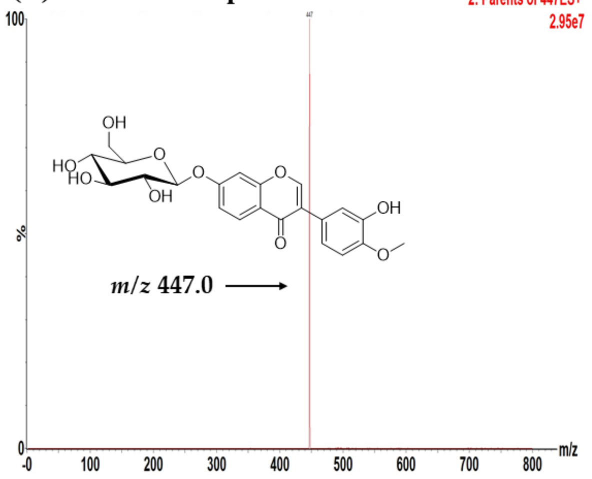

(D)-Product ion peak

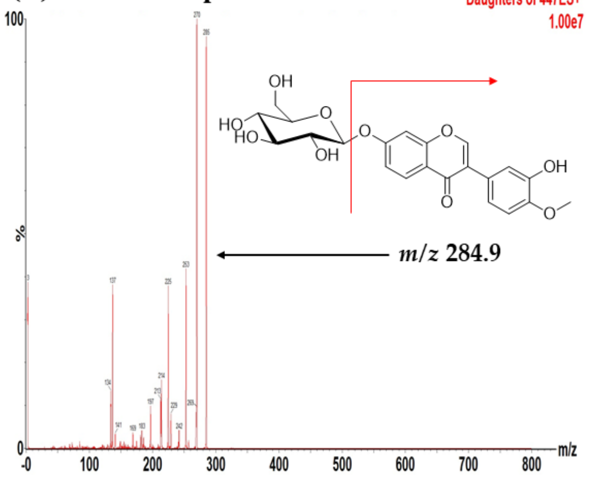

(E)-Precursor ion peak

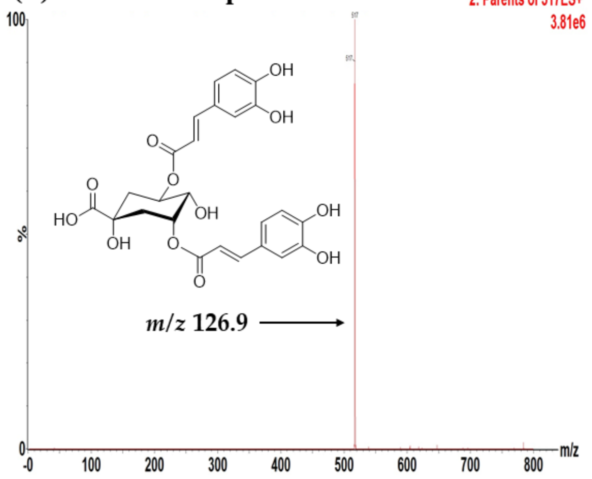

(E)-Product ion peak

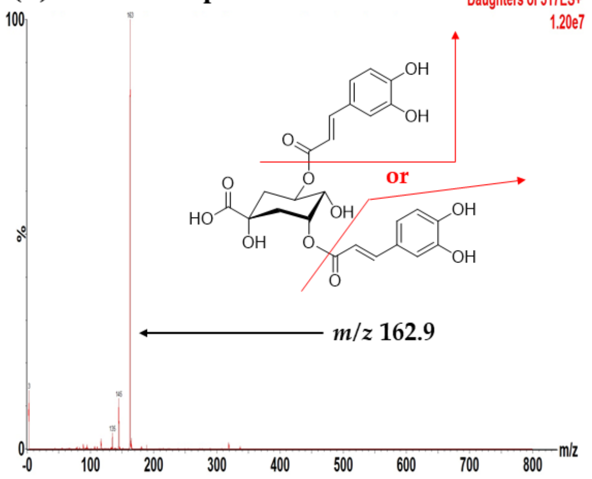

(F)-Precursor ion peak

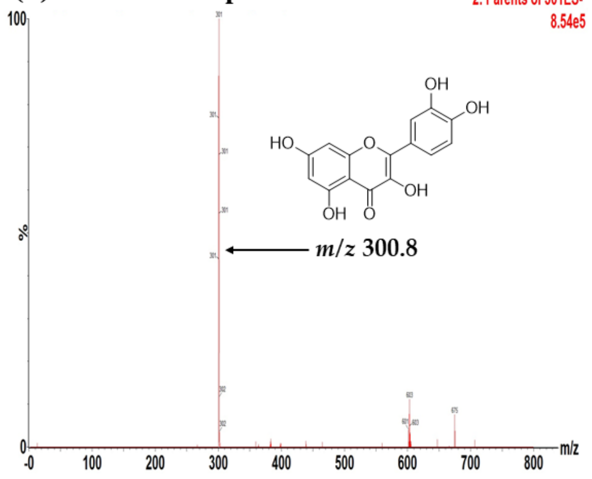

(F)-Product ion peak

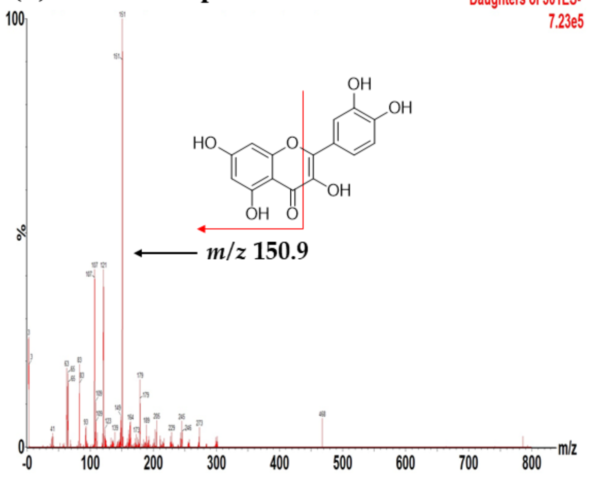

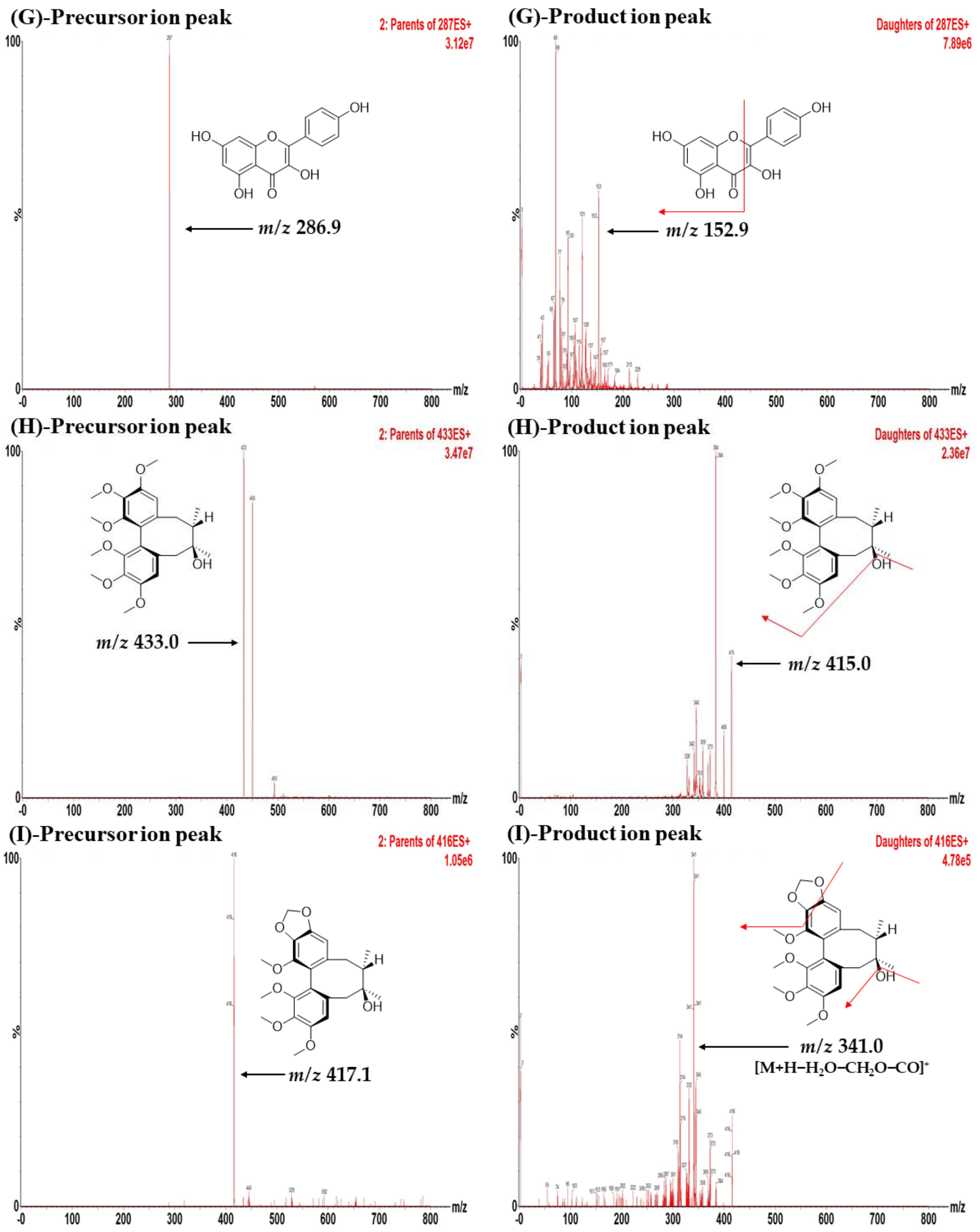

Figure S7. Precursor ion (Q1) and product ion (Q3) peaks for each target compound. Hydroxymethylfurfural (A), mulberroside A (B), chlorogenic acid (C), calycosin-7-*O*-glucoside (D), 3,5-dicaffeoylquinic acid (E), quercetin (F), kaempferol (G), schizandrin (H), and gomisin A (I).
